# Understanding protein diffusion on force-induced stretched DNA conformation

**DOI:** 10.3389/fmolb.2022.953689

**Published:** 2022-12-05

**Authors:** Anupam Mondal, Arnab Bhattacherjee

**Affiliations:** School of Computational and Integrative Sciences, Jawaharlal Nehru University, New Delhi, India

**Keywords:** DNA stretching, Σ-DNA, S-DNA, triplet formation, facilitated diffusion, protein−DNA interactions

## Abstract

DNA morphology is subjected to environmental conditions and is closely coupled with its function. For example, DNA experiences stretching forces during several biological processes, including transcription and genome transactions, that significantly alter its conformation from that of B-DNA. Indeed, a well-defined 1.5 times extended conformation of dsDNA, known as Σ-DNA, has been reported in DNA complexes with proteins such as Rad51 and RecA. A striking feature in Σ-DNA is that the nucleobases are partitioned into triplets of three locally stacked bases separated by an empty rise gap of ∼5 Å. The functional role of such a DNA base triplet was hypothesized to be coupled with the ease of recognition of DNA bases by DNA-binding proteins (DBPs) and the physical origin of three letters (codon/anti-codon) in the genetic code. However, the underlying mechanism of base-triplet formation and the ease of DNA base-pair recognition by DBPs remain elusive. To investigate, here, we study the diffusion of a protein on a force-induced stretched DNA using coarse-grained molecular dynamics simulations. Upon pulling at the 3′ end of DNA by constant forces, DNA exhibits a conformational transition from B-DNA to a ladder-like S-DNA conformation *via* Σ-DNA intermediate. The resulting stretched DNA conformations exhibit non-uniform base-pair clusters such as doublets, triplets, and quadruplets, of which triplets are energetically more stable than others. We find that protein favors the triplet formation compared to its unbound form while interacting non-specifically along DNA, and the relative population of it governs the ruggedness of the protein–DNA binding energy landscape and enhances the efficiency of DNA base recognition. Furthermore, we analyze the translocation mechanism of a DBP under different force regimes and underscore the significance of triplet formation in regulating the facilitated diffusion of protein on DNA. Our study, thus, provides a plausible framework for understanding the structure–function relationship between triplet formation and base recognition by a DBP and helps to understand gene regulation in complex regulatory processes.

## 1 Introduction

Protein–DNA interactions are central to several biological processes, such as DNA replication, transcription, repair, chromatin compaction, and gene regulation. Many of these processes can exert forces on DNA, such as the forces produced by DNA/RNA polymerases, as it moves over the template (Yin et al., 1995; Maier et al., 2000; Wuite et al., 2000), or the forces stemming from DNA itself for purposes including transport and tertiary structure manipulation (Nicklas, 1988). These pico-Newton (pN) scale forces exerted on DNA can affect the non-specific binding of proteins with the DNA. For instance, a range of forces applied to DNA has shown to greatly influence the binding of many DNA-binding proteins (Dahlke and Sing, 2018). Forces on DNA are also found to alter the dissociation rates of proteins from DNA (Xiao et al., 2010; Marko and Siggia, 1997; Marko, 2015) of ligands from receptors (Wu et al., 2007) and the DNA unwinding activity of hRPA protein (De Vlaminck et al., 2010). Furthermore, the mechanical properties of DNA under an applied force can potentially influence the DNA–protein interactions (Gore et al., 2006; Gemmen et al., 2006; Allemand et al., 2003). While these observations suggest that forces on DNA exist in a wide range of cellular processes, how it affects the protein–DNA recognition process remains completely unknown.

DNA can exist in various conformations under both *in vivo* and *in vitro* conditions, even in the absence of force (Alberts et al., 2008). The presence of external force alters the structure of DNA and exhibits multiple structural polymorphisms with a change in its helical parameters (Dans et al., 2012). Single-molecule pulling experiments (such as atomic force microscopy (AFM) (Clausen-Schaumann et al., 2000; Liu et al., 2010; Rief et al., 1999), optical/magnetic tweezers (Smith et al., 1996; Gosse and Croquette, 2002; Paik and Perkins, 2011) , and molecular dynamics simulations (Lebrun and Lavery, 1996; Kosikov et al., 1999; Harris et al., 2005; Garai et al., 2017; Luan and Aksimentiev, 2008) revealed that when canonical B-form DNA is pulled mechanically by external stretching forces, the double-stranded DNA (dsDNA) is extended to almost 1.7 times beyond its original contour length. It has been observed that DNA undergoes an abrupt overstretching transition from B-DNA to an overstretched conformation when pulled with forces of 60–70 pN and eventually denatures at relatively higher forces (Smith et al., 1996; Bosaeus et al., 2012). The force-extension curve of dsDNA shows a characteristic plateau that indicates a highly cooperative transition from B-DNA to an overstretched ladder-like S-DNA conformation (Cluzel et al., 1996; Luan and Aksimentiev, 2008). The structure of S-DNA is usually a partly untwisted ladder in which the base-pairing is preserved, but the average rise per base-pair is increased as compared to unstretched B-DNA. The overstretched conformation of DNA may play an important role in the function of proteins that are able to make use of these structural properties of DNA. For example, the X-ray crystal structure of RecA protein bound to the genomic DNA reveals a superhelical topology in which the dsDNA is underwound and stretched to approximately 50% in length relative to canonical B-DNA (Chen et al., 2008). Such a nucleoprotein complex, thus, presents a situation where the conformational change in DNA can be coupled with an external force. A striking observation in it is that the extended DNA adopts a configuration where the nucleobases are partitioned into orderly stacked triplets of base-pairs separated by a large rise gap. This form of DNA structure is physically plausible when considered purely in static chemical terms (Bertucat et al., 1998) and is previously referred (Bosaeus et al., 2017; Taghavi et al., 2017) to as “Σ-DNA,” which may be considered a special case of overstretched S-form DNA. The degree of extension observed in Σ-form DNA has been identified even in the absence of recombinase proteins by using single-molecule force spectroscopy experiments (Bosaeus et al., 2012) and considered the most stable conformation without any cofactors. Even the presence of DNA-binding cofactors or intercalators has been observed to form a triplet kind of stable disproportionate DNA structure in solution through atomistic simulations of duplex DNA under an applied force (Taghavi et al., 2017). In a recent study, Bosaeus et al. (2017) analyzed the possible biological role of Σ-phase DNA and speculated that partitioning of bases into triplets separated by a stack-breaking gap may have a connection with the physical origin of the existence of three letters (codon/anti-codon) in the genetic code. Thus, one may ask whether the structural distortion in Σ-DNA is just a coincidence of protein binding or somehow the appearance of these structures is related to any biological function. Does this triplet structures have any connection in enhancing the recognition of base sequences by DNA-binding proteins? How do these structural changes in DNA under tension affect its interaction with the protein?

In order to understand these issues, we studied protein diffusion on force-induced stretched DNA using coarse-grained molecular dynamics simulations. Previously, many studies have probed the diffusion of a DNA-binding protein on unstretched DNA, in particular, the target search process on DNA by proteins. Several experimental (Blainey et al., 2009; Iwahara and Clore, 2006a, Iwahara and Clore, 2006b; Esadze and Iwahara, 2014; Elf et al., 2007; Clore, 2011), simulation (Marklund et al., 2013; Guardiani et al., 2014; Mondal and Bhattacherjee, 2015; Givaty and Levy, 2009; Bhattacherjee et al., 2016; Bhattacherjee and Levy, 2014a, Bhattacherjee and Levy, 2014b), and theoretical (Kolomeisky and Veksler, 2012; Mirny et al., 2009; von Hippel and Berg, 1989; Veksler and Kolomeisky, 2013; Mondal and Bhattacherjee, 2017) studies suggest that proteins associate their target sites orders of magnitude faster than a simple 3D diffusion-limited process. The mechanism behind this rapid translocation of proteins is due to the idea of facilitated diffusion, where the protein exhibits 1D diffusion along DNA in combination with 3D diffusion in the bulk. In 1D events, when the protein is associated non-specifically with DNA, it can move stochastically from one base to another along the contour of DNA (sliding). When detached from the DNA surface, protein can re-associate to a nearby site (hopping) after a short time period or can jump to a distant DNA segment (intersegmental transfer) (Mondal et al., 2022b). In our study, we pulled the DNA molecule by applying constant force to the 3′-3′ end of DNA and observed that DNA undergoes a conformational transition from B-DNA to an overstretched ladder-like S-DNA conformation *via* an intermediate Σ-DNA transition. Our analysis suggests that the resulting stretched DNA conformations feature non-uniform clusters of nucleotide bases such as doublets, triplets, and quadruplets separated by a large rise gap. Among these base-pair clusters, triplets are more stable than others, which protein favors to form during its 1D translocation along DNA, and the relative population of base-pair triplets governs the ruggedness of the protein–DNA binding energy landscape and thereby the ability of a protein to locate its target DNA sites. By analyzing different force regimes, we delineate the underlying translocation mechanism of a DNA-binding protein and underscore the significance of triplet formation in regulating the protein diffusion on stretched DNA.

## 2 Materials and methods

### 2.1 Protein and DNA model

In this work, we adopt coarse-grained models of protein and DNA to study protein diffusion along force-induced stretched DNA. The protein is represented by one bead per amino acid located at the respective *C*_*α*_ position (Bhattacherjee et al., 2016). The energetic of the protein is described by a structure-based Lennard–Jones potential that ensures the formation of native contacts found in the folded structure of the protein (Clementi et al., 2000b). Such a reduced model promises great advantages in studying the funnel-like energy landscape of protein folding (Clementi et al., 2000a), protein–protein interactions (Bhattacherjee and Wallin, 2012) , and multiple basins free energy landscape for large-scale motion of proteins (Okazaki et al., 2006). For DNA, we use a 3SPN.2C coarse-grained model developed by de Pablo’s group in which each nucleotide is described by three beads and is positioned at the geometric centers of the phosphate, sugar, and base atoms (Freeman et al., 2014a). The 3SPN.2C model accurately estimates the correct structural properties of DNA such as helix width, base-pair rise, number of base-pair per turn, major and minor groove widths which are in good agreement with experimental results (Hinckley et al., 2013). The model successfully reproduces the mechanochemical properties of DNA such as sequence-dependent persistence length and flexibility of double-stranded DNA, prediction of melting temperature, and estimation of DNA hybridization rate constants for varying sequences under different ionic concentrations. Most importantly, the 3SPN.2C DNA model is developed by Freeman et al. (2014a) with the intention of studying interactions between protein and dsDNA only, and a special emphasis is placed on successfully capturing the effects of sequence-dependent DNA shape, local flexibilities, dsDNA persistence lengths, melting temperatures, and minor groove width profiles, which are extensively validated against experimental data. By incorporating these relevant DNA properties, the authors have recently identified the structural properties most critical to nucleosome formation (Freeman et al., 2014b) and found that histone– DNA binding affinity is encoded in its sequence-dependent shape, including subtle deviations from the ideal linear B-DNA geometry. Indeed, previous studies support that even nuances in the groove width and local variations in minor groove width play a significant role in its interaction with the proteins (Bhattacherjee and Levy, 2014a). A similar DNA model (3SPN.2C) has been extensively used previously by us (Mondal et al., 2022a, Mondal et al., 2022b) and other groups (Mondal et al., 2022a, Mondal et al., 2022b; Nagae et al., 2021; Brandani et al., 2021; Lin et al., 2021; Tan and Takada, 2020; Moller et al., 2019; Lequieu et al., 2017; Niina et al., 2017; Lequieu et al., 2016) to demonstrate the role of DNA shape dynamics and flexibility in regulating the interactions with proteins.

### 2.2 Protein–DNA interactions

Interactions between protein and DNA molecules are treated at a non-specific level and are driven by electrostatic interactions and excluded volume effects. Excluded volume interactions are modeled by an *r* ^12^ repulsive potential that penalizes the steric clashes during the non-specific encounter between them over the simulation. The electrostatic interactions between positively and negatively charged residues of protein and DNA are modeled by the Debye–Hückel potential. A negative charge of −0.6 is assigned to each phosphate atom to take the counterion condensation effect, and a net charge of +1 is assigned to lysine (Lys) and arginine (Arg) and − 1 for glutamate (Glu) and aspartate (Asp) residues. It is to note that the Debye–Hückel theory provides the advantage of introducing the effect of salt concentrations in the present model and thus enables us to investigate protein diffusion on double-stranded DNA in an ionic environment. Although the theory is limited to only dilute salt concentration, previously, the Debye–Hückel theory has been successfully applied to shed light on many crucial aspects of protein–DNA interactions (Mondal and Bhattacherjee, 2015; Bhattacherjee and Levy, 2014a, Bhattacherjee and Levy, 2014b; Dey and Bhattacherjee, 2018, Dey and Bhattacherjee, 2019a, Dey and Bhattacherjee, 2019b, Dey and Bhattacherjee, 2020; Mondal et al., 2022a, Mondal et al., 2022b).

### 2.3 DNA stretching

The DNA molecule is subjected to constant stretching forces −*F* and +*F* applied to the 3′ termini. The stretching force is kept fixed during the simulation to investigate the large-scale motion of protein diffusion on a stretched DNA. The value of the constant stretching force varied in the range of 0–200 pN. A zero value of stretching force signifies that there is no external force acting on DNA. The DNA molecule is stretched along the Z-direction, and therefore, the energy function used for DNA stretching is given as follows: $E_{\mathrm{stretch}}=-\vec{F}.\vec{dz}$ , where $\vec{dz}$ is the DNA end-to-end extension.

### 2.4 Simulation protocol

To probe the dynamics of protein search on DNA, we choose Sap-1 protein from Protein Data Bank (PDB ID: 1BC8) as the searching protein. This DNA-binding protein is a 93-residue long globular protein consisting of 15 positively and six negatively charged amino acids and contains a winged-helix recognition region to scan DNA. The starting sequence-dependent structure of 100-bp B-DNA is generated from X3DNA (Zheng et al., 2009). The DNA sequence is given in Supplementary Figure S1 that contains 67% GC-content and 33% AT-content. The choice of this GC-rich sequence is inspired by the recent experimental studies (Bosaeus et al., 2012, Bosaeus et al., 2017). Both the molecules are placed at the center of a simulation box of dimension 350 Å × 350 Å × 800 Å. DNA is placed along the Z-axis, and the periodic boundary conditions are applied in all the directions. Such box dimensions are chosen in order to ensure that fully extended ( ∼80%−100% stretched compared to its initial length) DNA can also be placed within it. We then apply a constant pulling force ranging from 0 to 200 pN on both the 3′ ends of DNA and study the protein diffusion on force-induced stretched DNA in a physiological salt concentration of 140 mM. While doing so, it is to be noted that during the simulation, only DNA is stretched, not the protein. Initially, protein is placed 40 Å away from the DNA surface along the X-direction. The time evolution of the system is governed by Langevin dynamics at temperature 300 K and friction coefficient *γ* = 0.05 kg/s. At every constant pulling force (0–200 pN), we perform 40 independent simulations, each 1 × 10^8^ MD steps long, in the canonical ensemble with the Langevin thermostat. The initial 0.4 × 10^8^ MD steps are discarded to ensure thermal equilibrium, after which all the analyses are performed in the present study.

## 3 Results and discussion

### 3.1 Force–strain response of dsDNA

**FIGURE 1:**
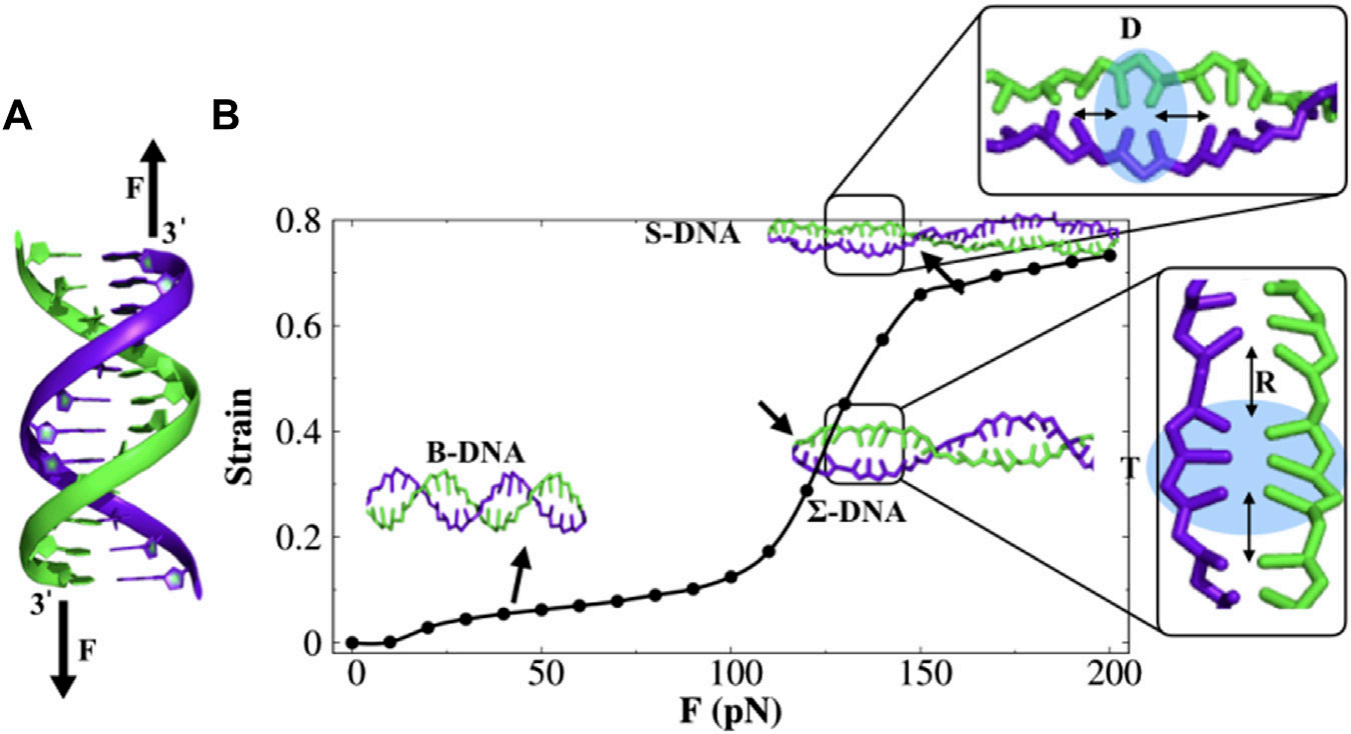
Double-stranded DNA (dsDNA) under constant stretching force. **(A)** Force is applied to the 3′ ends of both the strands, shown by black arrows. **(B)** Force–strain curve of dsDNA. With the increase in force, dsDNA undergoes a structural transition from B-DNA to S-DNA *via* an intermediate transition, referred to as Σ-DNA. The highlighted region in both the S-DNA and Σ-DNA conformations shows the appearance of stacked doublets (D) and triplets (T) of nucleobases separated by an empty gap of length *R*. The error bar for each symbol is defined as the standard error. The associated error bars are smaller than the point size.

In order to understand the structure and dynamics of force-induced stretched DNA, we first probe the force–strain response of dsDNA. We apply constant stretching/pulling force on 3′ termini of the two strands of a randomly selected 100-bp GC-rich B-DNA molecule (see Figure 1A) containing 67% GC-content and 33% AT-content and perform coarse-grained molecular dynamics (MD) simulations. The constant stretching force is varied from 0 pN to 200 pN, and we present the force vs. strain curve in Figure 1B, where the strain is the ratio of the change in the end-to-end distance of the 3′-3′ ends of the two strands to its equilibrium contour length. We find that the stretching behavior of dsDNA in Figure 1B can be divided into three regimes. At forces *F* < 110 pN, DNA gets stretched by 10%–15% to its initial contour length. In this force regime, the slope of the curve has increased slightly, and DNA exists in its B-form conformation. However, there is a sharp increase in the slope from 110 pN up to about 150 pN where dsDNA starts to deform its helical structure (see Supplementary Video S1) and achieves 20%–60% extension compared to B-DNA and adopts a special kind of conformation referred to as Σ-DNA, which is stretched to about 50% in length compared to canonical B-DNA. Such a degree of extension is found in DNA complexed with recombinase proteins RecA and Rad51 (Chen et al., 2008; Xu et al., 2017). Indeed, a similar degree of extension is also observed in force-induced stretched GC-rich duplex DNA molecules (Bosaeus et al., 2012, Bosaeus et al., 2017). It is, however, known that the nature of the overstretching transition depends critically on the nature of the bp composition. For instance, in the experimental study of Bosaeus et al. (2012) and Bosaeus et al. (2017), the authors have chosen 60–64-bp short DNA molecules with base sequences containing 60% GC-content and 70% AT-content and observed that when the AT content is high (70%), the DNA molecule tends to overstretch *via* denaturation. Therefore, AT-rich sequences are not desirable for studying protein diffusion on stretched DNA. In contrast, GC-rich sequences (60% GC) are found to undergo a conformational transition from B-DNA to a distinct form of overstretched DNA that is characterized by a 51% extension and remains base-paired. Higher-level coarse-grained modeling studies (Manghi et al., 2012) tend to confirm this scenario. This observation suggests that the DNA sequence is an important parameter for controlling the transition from B-form to stretched and/or melted forms. In the present study, we consider a DNA sequence with the GC-content comparable to the one used in previous experimental studies to specifically investigate the protein diffusion on stretched DNA.

Furthermore, increasing the force (above 150 pN) would lead to a plateau region, where the dsDNA extended 70%–80% from its initial contour length and emerged a ladder-like overstretched conformation, the so-called S-DNA. Previously, the existence of such an S-DNA structure has been found using experimental (Smith et al., 1996; Cluzel et al., 1996) and theoretical studies (Lai and Zhou, 2003). Some typical snapshots of different DNA structures at various stretching forces are provided in Supplementary Figure S2. We also monitored the changes in DNA length during the simulation and evaluated the kinetics of the structural transition between different forms of DNA at individual forces. The results are presented in Supplementary Figure S3. The result shows that the structural transition appears much faster with an increasing stretching force. For instance, the transition from B-DNA to Σ-DNA (50% extension) is ∼ 12.4 times faster at force *F* = 180 pN than that at force *F* = 140 pN, and the transition from Σ-DNA to S-DNA (70% extension) appears to be ∼ five times faster at *F* = 190 pN than that at *F* = 170 pN.

The overall behavior of the force–strain curve is consistent with the experimentally observed force–strain curve reported previously (Smith et al., 1996; Cluzel et al., 1996; Danilowicz et al., 2009). It is interesting to note that the bases in the overstretched conformation of DNA are organized in the form of doublets, triplets, quadruplets, and so on, followed by a rise gap of length *R* (see Figure 1B). Recently, the existence of a cluster of three bases (triplet) in Σ-DNA was reported, and the possible biological role of it was explained (Bosaeus et al., 2017). The formation of base triplets has been hypothesized to be correlated with the origin of a genetic code (Bosaeus et al., 2017). This raises a question of whether such triplets promote easier access to the DNA base-pairs by DNA-binding proteins (DBPs).

### 3.2 Characteristic details of base-pair clusters

**FIGURE 2:**
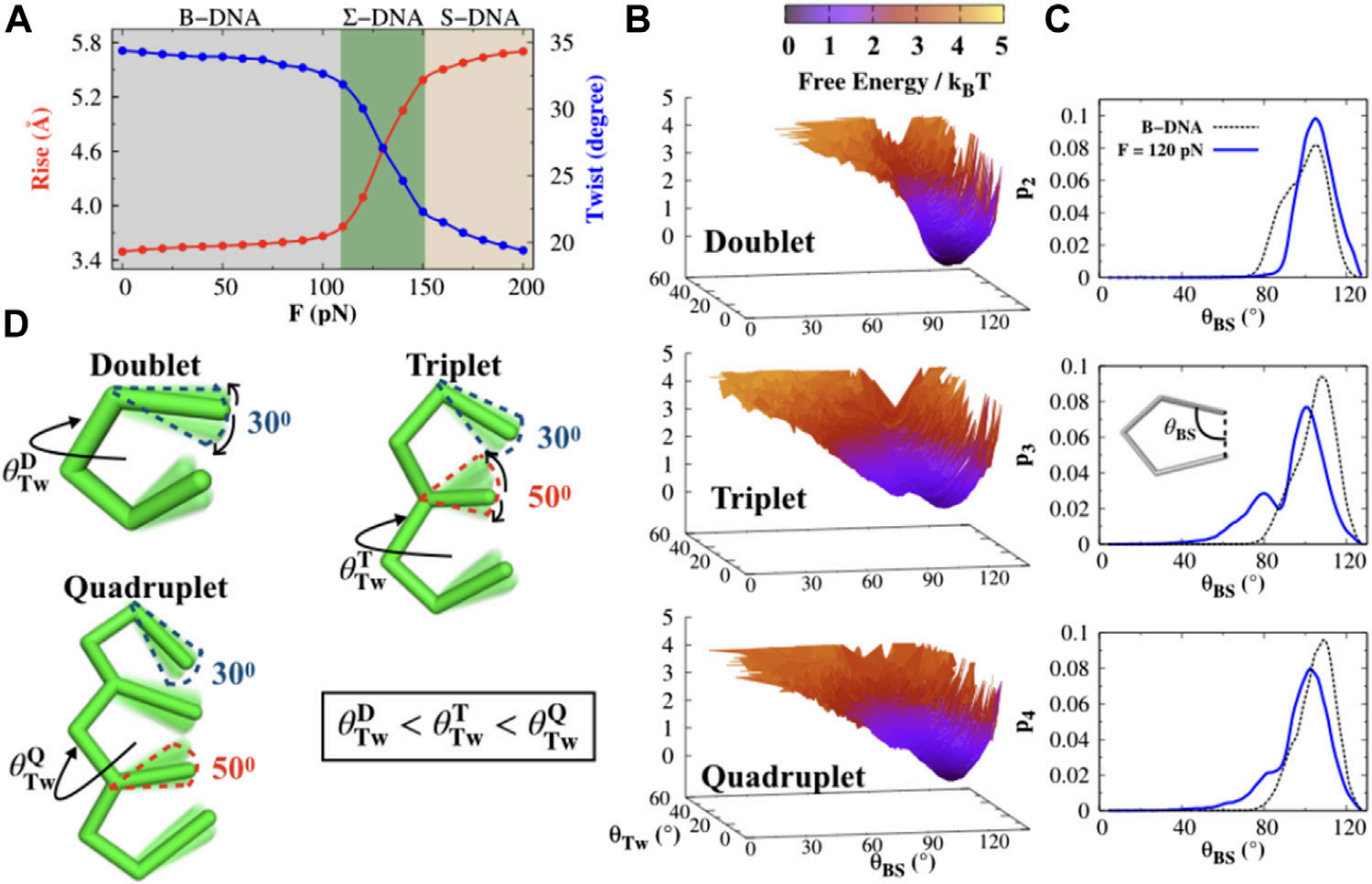
Structural characterization of the DNA bases. **(A)** DNA helical rise and twist angles are calculated as a function of stretching force F. The three shaded regions gray, green, and yellow correspond to three different DNA conformations, namely, B-, Σ-, and S-DNA that emerge due to stretching. The error bar for each symbol is defined as the standard error. The associated error bars are smaller than the point size. **(B)** Free energy profiles *F*(*θ*_*Tw*_, *θ*_*BS*_) for the doublet, triplet, and quadruplet at force *F* = 120 pN, where *θ*_*Tw*_ and *θ*_*BS*_ are the helical twist and base-stacking angles. **(C)** Probability histograms of the base-stacking angles (*θ*_*BS*_) for the doublet (*p*_2_), triplet (*p*_3_), and quadruplet (*p*_4_) at a stretching force *F* = 120 pN (blue solid line) compared with B-DNA (dashed line). **(D)** Schematic representation of the structure of the doublet, triplet, and quadruplet. The twist angles for the doublet $\left(\theta_{Tw}^{D}\right)$ , triplet $\left(\theta_{Tw}^{T}\right)$ , and quadruplet $\left(\theta_{Tw}^{Q}\right)$ are shown by black arrows that follow a relation specified in the rectangular box. The fluctuation in each base for the doublet, triplet, and quadruplet is shown by a blurred motion view. The range of angles of the bases that arise due to fluctuation is displayed individually.

Before proceeding with investigating the same, we first characterize the structural details of the base-pair clusters under constant stretching force. In Figure 2A, we consider the two helical parameters, namely, the helical rise and DNA twist angle, and present their variation under constant stretching force. At forces *F* < 110 pN, DNA remains in its B-form structure, where the average rise ( ∼3.5 Å) and twist (∼34.0°) remain constant. In the intermediate force regime (from 110 pN to 150 pN, green shaded region), a structural change from B-DNA to Σ-DNA causes a sharp increase in the helical rise from ∼3.80 Å to ∼5.38 Å and a rapid drop in the twist angle from ∼32° to ∼22° . This is because the stretching of B-DNA leads to an increase in the distance between consecutive bases and the unwinding of duplex helices. When S-DNA emerges (*F* > 150 pN), the helical rise reaches a plateau and the twist angle decreases up to 20°. The average rise per base-pair in S-DNA reaches a new value of ∼5.8 Å, which is consistent with the value reported recently (Taghavi et al., 2017). This suggests that our coarse-grained DNA model is suitably amalgamated under constant stretching force and precisely captures the correct two-state transition from B-DNA to a partly untwisted ladder-like S-DNA structure *via* an intermediate conformation Σ-DNA.

Having seen the correct two-state transition, we observe that the main variation in the rise and twist angle arises when dsDNA adopts the Σ-form conformation. In this transition, the backbone of DNA gets extended by about 50% in length, which favors the formation of base-pair clusters such as doublets, triplets, and quadruplets. Due to the formation of such base-pair clusters, the consecutive groups of stacked bases are separated (by ∼5.0 Å) non-uniformly and interrupt the stacking interactions. The stacking energy for the doublet, triplet, and quadruplet is calculated as − 3.01 ± 0.001 kcal/mol, − 3.06 ± 0.001 kcal/mol, and − 2.93 ± 0.002 kcal/mol, respectively. This suggests that the triplet is more stable than doublet and quadruplet arrangements, which is consistent with the result of Bosaeus et al. (2017). In Figure 2B, we estimated the two-dimensional free energy profiles of the doublet, triplet, and quadruplet at a constant force (*F* = 120 pN) in which Σ-DNA appears and is presented as a function of twist and base-stacking angles. Our results show distinctly different free energy surfaces for doublet, triplet, and quadruplet formation. A single minimum is observed in the free energy surface of the doublet. In contrast, the free energy surface of the triplet and quadruplet shows two distinct minima. The corresponding probability histogram for forming the doublet (*p*_2_), triplet (*p*_3_), and quadruplet (*p*_4_) is shown in Figure 2C as a function of the base-stacking angle (*θ*_*BS*_), which are then compared with the case for ideal B-DNA geometry, i.e., in the absence of stretching force. The base-stacking angle for doublets shows a variation between 90° and 120°, which suggests that the nucleobases in a doublet can fluctuate around 30° (90°–120°) during stacking interactions (see Figure 2D). Unlike doublets, the probability distribution for triplets and quadruplets features a bimodal distribution for base-stacking angles. Among the two modes in the distribution, the major mode varies between 90° and 120°, whereas the variation in the minor mode lies within 40°–90°. The corresponding fluctuation in the base-stacking angle for the nucleobases in triplets and quadruplets is shown in Figure 2D. It is interesting to note that the base-stacking angle in Σ-DNA obtained from our simulation agrees well with the values calculated from the crystal structure of the RecA–dsDNA filament (Chen et al., 2008) (see Supplementary Figure S4). Furthermore, depending on the type of base-stacking and base-pairing interactions, we observed different local conformations in Σ-DNA. For example, we find five different local conformations of the base-pair step of the sequence GG ⋅ CC, which are then classified as base-pair stacked, base-pair tilted, base-pair no stack, and melted and mismatched conformations (see Supplementary Figure S5). All these local conformations match very well with the local conformations obtained from the all-atom simulation of Taghavi et al. (2017) having the same base-step sequence. Taken together, the consistency between our results and previous studies (Taghavi et al., 2017; Chen et al., 2008) in capturing the characteristic details of base-pair clusters validates our model and provides confidence to use the same and investigate if such base-pair clusters facilitate easier access to the DNA bases to DBPs. Perhaps, a plausible way to answer the question is to study interactions of a DBP on stretched DNA conformation and probe how efficiently the protein may scan the DNA bases.

### 3.3 Protein favors the formation of base-pair triplets under constant stretching force

**FIGURE 3:**
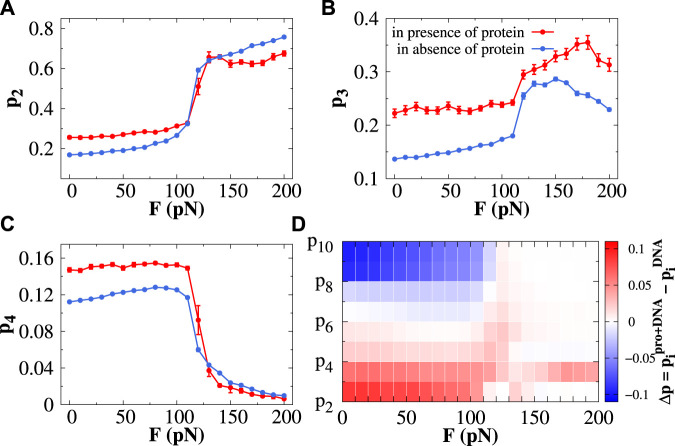
Effect of protein dynamics in the formation of base-pair clusters. The probability of forming the **(A)** doublet (*p*_2_), **(B)** triplet (*p*_3_), and **(C)** quadruplet (*p*_4_) is plotted as a function of stretching force in the presence (red line) and absence (blue line) of protein. The error bar for each symbol is defined as the standard error. **(D)** Presented contour plot combines the probability of forming all kinds of clusters starting from the doublet (*p*_2_) and triplet (*p*_3_) to decuplet (*p*_10_) with respect to stretching force, where the color bar represents the differences in the probabilities of forming different clusters in the presence and absence of protein.

We select Sap-1 protein as a representative DNA-binding protein since it has been widely studied both computationally (Bhattacherjee and Levy, 2014a; Mondal and Bhattacherjee, 2015; Dey and Bhattacherjee, 2018, 2019a, 2020) and experimentally (Mo et al., 1998; Guan et al., 2017) and investigate what effect does it bring on the formation of base-pair clusters while diffusing on stretched DNA. In Figure 3, we estimate the probability of forming doublets (*p*_2_), triplets (*p*_3_), and quadruplets (*p*_4_) in the presence and absence of Sap-1 protein as a function of constant stretching force *F* ranging from 0 to 200 pN. The same for higher-order base-pair clusters is shown in Supplementary Figure S6. In the presence of Sap-1 protein, the doublets (or triplets or quadruplets) are identified according to the following condition: first, we identify the closest DNA base-pair *i* from the recognition region of the Sap-1 protein when it diffuses one-dimensionally along the DNA surface and consider the segment (*i* − 6, *i* + 6). Within this segment, if the two (or three or four) consecutive nucleobases are separated by an empty gap of length 5 Å, then it will be considered forming a doublet (or triplet or quadruplet). The DNA segment (*i* − 6, *i* + 6) is particularly chosen, mainly because of the fact that a DNA segment spanning *i* − 6 to *i* + 6 around the protein-binding site covers a complete helical turn of 10.5 bp along DNA. Sap-1 protein binds specifically with a 9-bp short DNA stretch (almost a complete helical turn) through its recognition helix inside the DNA major groove. The choice, therefore, directly allows us to track the formation of base-pair clusters (from doublets to decuplets) along DNA when the protein scans the DNA bases through sliding/hopping modes, i.e., one-dimensionally. The same analysis performed on the protein-excluded region, i.e., the region other than the segment (*i* − 6, *i* + 6), is considered for analyzing the formation of different base-pair clusters in the absence of the protein. Following this procedure, we counted the number of doublets, triplets, and so on from the whole simulation trajectory, and the corresponding probability is calculated from the definition prescribed in the Supplementary Material. At forces *F* < 110 pN, protein favors the formation of doublets, triplets, and quadruplets compared to its unbound form (Figures 3A–C), and a reverse trend is observed for higher-order base-pair clusters (Supplementary Figure S6). When Σ-DNA and S-DNA emerge (*F* > 110 pN), we observe that only *p*_2_ and *p*_3_ increase with increasing *F* and then saturate (Figures 3A,B). Other probabilities (from *p*_4_ to *p*_10_) exhibit a decreasing trend with *F* (Figure 3C and Supplementary Figure S6), mainly because of the too-high backbone strain for higher-order base-pair clusters. Another important observation in this force regime is that the triplet formation propensity is facilitated by the presence of Sap-1 protein compared to its unbound form (Figure 3B). This feature is observed only for triplets (and not in doublets or quadruplets) and is the most important result of our study. Also, it is worthwhile to mention here that the doublet propensity at forces *F* > 110 pN is much higher than the triplet propensity; however, in comparison to its unbound form, the protein favors the base-pair triplets to form in this force regime while diffusing one-dimensionally along DNA. In Figure 3D, we have shown a plot that combines the probability of all different base-pair clusters (from *p*_2_ to *p*_10_) with respect to *F*. The color bar represents the differences in the probability in the presence and absence of the protein. Having seen the analysis clearly indicating that the formation of base triplets is favored by the diffusing protein relative to its unbound form under constant stretching force, we next move to explore the impact of such base clusters in regulating the accessibility of DNA bases to the searching protein.

### 3.4 Base-pair triplets reduce the ruggedness of the potential energy landscape and enhance the protein diffusivity

**FIGURE 4:**
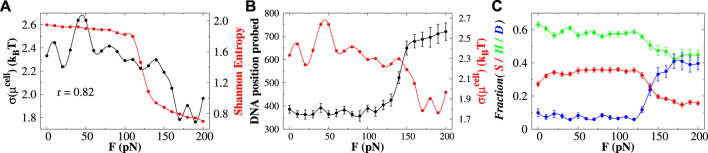
Effect of base-pair clusters on the diffusion of protein. **(A)** Correlations between the Shannon entropy *H* (denoted by the red line) and the roughness (standard deviation of *μ* ^*cell*^) of the chemical potential landscape (indicated by the black line) with the stretching force. The value of *r* indicates the correlation coefficient between them. **(B)** DNA position probed (black line) and the roughness of the chemical potential (red line) as a function of stretching force. **(C)** Effect of stretching force on the interplay between sliding (S), hopping (H), and 3D diffusion (D) by the searching protein. The error bar for each symbol is defined as the standard error. In **(A)** and the red curve in **(B)**, the associated error bars are smaller than the point size.

To address the issue, we measure the heterogeneity of DNA base-pair clusters by estimating the Shannon entropy as a function of stretching force *F* from the associated probabilities of different clusters (see Figure 4A). The Shannon entropy (*H*) is defined as

$$H=-\underset{i=2,3,\ldots ,10}{\sum }p_{i}\;\log\;p_{i}.$$ (1)

Eq. 1 gives information about the distribution of the base-pair clusters—whether they occur with an equal probability (high entropy value) or whether some other clusters prevail (low entropy value). It, however, does not reflect the correlations between different base-pair clusters. At forces *F* < 110 pN, the Shannon entropy values are high and remain roughly constant, suggesting similar probability distribution of different base-pair clusters. At forces *F* > 110 pN (when both Σ-DNA and S-DNA arise), the *H*-value sharply decreases, indicating that the formation of some base-pair clusters prevails. Since the probability of doublets and triplets shows a higher probability (Figure 3) among which protein favors the formation of base triplets compared to its unbound form in this force regime, the information on the formation of base-pair triplets might have some connection with the diffusivity of the searching protein. To quantify the effect, we estimate the ruggedness of the potential energy landscape by measuring its roughness. We follow the method prescribed by Putzel et al. (2014) and consider the position of the center of mass of the recognition region of the Sap-1 protein as the order parameter. We partition the space into cubic cells of size 50 Å^3^ and measure the probability of the cell *i* to be occupied by the diffusing protein. As the searching protein moves between different cells, it will experience an effective potential equal to the excess contribution to the diffusing site’s chemical potential $\left(\mu_{i}^{\mathrm{cell}}\right)$ :

$$\mu_{i}^{\mathrm{cell}}=-k_{B}T\;.\;\ln\left(V_{\text{cell}}^{-1}\int_{\text{cell}\;i}e^{-\beta U\left(\vec{r}\right)}d\vec{r}\right),$$ (2)

where $U\left(\vec{r}\right)$ is the total potential energy acting on the recognition site at position $\vec{r}$ . By discretizing the aforementioned integral (see Supplementary Text for details), the effective potential of each cell can be calculated, and consequently, the roughness of the landscape can be measured from the corresponding standard deviation *σ*(*μ* ^*cell*^). In Figure 4A, the roughness is plotted against the applied stretching force. We observe that with increasing force, the roughness of the potential energy surface decreases steadily. Interestingly, we found a strong correlation (*r* ∼ 0.82) between the ruggedness of the chemical energy landscape of the diffusing protein and the Shannon entropy of the base-pair clusters. As the protein favors triplets in the Σ-DNA and S-DNA regime compared to its unbound form, the formation of such base triplets reduces the ruggedness of the effective potential of the diffusing protein. We evaluate the impact of the ruggedness on the 1D diffusivity of Sap-1 protein by measuring the DNA search efficiency. In Figure 4B, we measure the DNA probed position, which indicates the number of DNA sites that are sampled by the searching protein through 1D diffusion only. In this measure, any DNA site that is visited by 1D diffusion (sliding + hopping, see Supplementary Text for criteria) is counted to the position probed measure regardless of whether it was already been scanned earlier in the same 1D cycle. When the protein gets dissociated from the DNA surface, the number of positions probed by the protein remains unchanged. The corresponding result in Figure 4B suggests that as the force increases, the less rugged the potential energy landscape, the more impactful is the search efficiency of DNA. This result is also supported by the measurement of the 1D diffusion coefficient *D*_1_ of the Sap-1 protein as a function of different stretching forces as shown in Supplementary Figure S7. Most importantly, the search efficiency sharply increases in the Σ-DNA regime (110 pN <F<150 pN) and reaches a maximum value when DNA exists in S-DNA conformation (*F* > 150 pN). Interestingly, in these force regimes, the protein facilitates the formation of base-pair triplets compared to its unbound form (Figure 3B), the relative population of which governs the ruggedness of the binding energy landscape of the protein and thereby promotes efficient searching of the protein on the stretched DNA track.

Having seen the diffusion efficiency, we now move to understand the non-specific target search mechanism of the searching protein. By analyzing the structural details of each snapshot generated during the simulations, we estimate the propensities of different translocation modes such as the sliding, hopping, and 3D diffusion as a function of the applied force *F*, and the results are presented in Figure 4C. The detailed criteria of each of these search modes are described in the Supplementary Text. Our result suggests that at forces *F* < 110 pN, the propensity of each search mode remains constant. In this force regime, the Sap-1 protein mostly performs sliding and hopping dynamics on DNA and performs less 3D diffusion (<10%) in the solution. The hopping mode in which the protein binds to DNA not so tightly as they do in the sliding mode, but still close to DNA, shows the highest population (∼60%) , whereas the sliding propensity exhibits ∼35% propensity to the overall search dynamics. In contrast, when Σ-DNA appears (110 pN <F<150 pN), 3D diffusion increases significantly (∼40%) , and both sliding and hopping propensities decrease simultaneously. At forces *F* > 150 pN (when S-DNA appears), each sliding, hopping, and 3D diffusion saturates to a value of ∼15%,45% , and 40%, respectively. To further understand the behavior of different search modes with varying *F*, we divided the forces into two regimes: a low-force regime ranging from 0 to 110 pN and a high-force regime ranging from 120 pN to 200 pN. We then investigate the mechanistic details of the non-specific search dynamics in these two force regimes.

### 3.5 Mechanistic details of protein search dynamics under low- and high-force regimes

**FIGURE 5:**
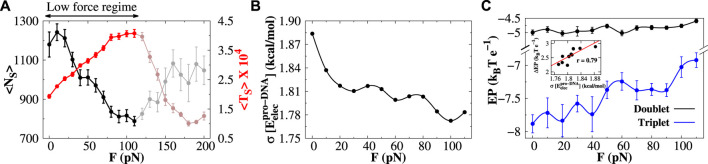
Protein diffusion under low stretching force. **(A)** Variations in the average number of sliding events ( $<N_{S}>$ ; black line) and the average time duration ( $<T_{S}>$ ; red line) of a sliding event are plotted as a function of stretching force. Only the regime under low stretching force is highlighted. **(B)** Fluctuation (standard deviation) in the protein–DNA electrostatic energy is shown with respect to stretching force. **(C)** Electrostatic potential of the DNA molecule is calculated at the reference points in the major groove as a function of *F* for doublets (black line) and triplets (blue line) using the Delphi program (Li et al., 2012). The correlation between the difference in the electrostatic potential for doublets and triplets and the fluctuation in the protein– DNA electrostatic energy is shown in the inset. The red line represents the best fits. The error bar for each symbol is defined as the standard error. In **(B)**, the associated error bars are smaller than the point size.

In Figure 5A, we present the average number of sliding events $<N_{S}>$ and their duration $<T_{S}>$ as a function of *F*. By the sliding event, we mean a time stretch during which Sap-1 continuously scans DNA through sliding dynamics only. The protein can perform hopping or 3D diffusion between two sliding events. A similar analysis for the variation in the average number of hopping events and their average time duration is presented in Supplementary Figure S8. In the low-force regime, the average number of sliding events decreases with *F*, and the corresponding time duration increases as the force increases. In contrast, both the parameters show an opposite trend in the high-force regime. To understand the rationale behind this, we first explain the behavior in the low-force regime (highlighted in Figure 5A). We find that the fluctuation in the protein–DNA electrostatic energy $\left(\sigma \left(E_{\mathrm{elec}}^{\mathrm{pro-DNA}}\right)\right)$ decreases in the low-force regime (Figure 5B). This is also supported by the result that the ruggedness of the protein–DNA binding energy landscape was found to have a decreasing pattern (Figure 4A and Supplementary Figure S9). What causes the reduced fluctuation of the electrostatic interactions between protein and DNA? Does this fluctuation have any direct connection with the formation of base-pair doublets or triplets? To investigate the same, we estimate the electrostatic potential (EP) along the DNA contour using the Delphi program (Li et al., 2012) by solving the non-linear Poisson–Boltzmann equation. The detailed procedure for the calculation of EP along DNA is described in the Supplementary Text. From the calculation, we identify the base-pairs that are involved in the formation of base-pair doublets and triplets along DNA by the searching protein and extract the values of the corresponding EP energies, and the results are presented in Figure 5C as a function of *F*. We observe that EP energies along the DNA contour for doublets are almost invariant with increasing *F*. However, the same for the triplets is found to have a variation of about 1 *k*_*B*_ *T* in the low-force regime. Since the EP energy for doublets is almost constant and for triplets, the EP energy varies with increasing *F*, the difference Δ*EP* of the EP energy for doublets and triplets will have a variation with *F*. Plotting the difference Δ*EP* with the fluctuation $\sigma \left(E_{\mathrm{elec}}^{\mathrm{pro-DNA}}\right)$ (see inset in Figure 5C), we find a ∼79% linear correlation between the two, suggesting that the formation of base-pair triplets reduces the $\sigma \left(E_{\mathrm{elec}}^{\mathrm{pro-DNA}}\right)$ . As a result, the protein can smoothly perform sliding dynamics on a less rugged DNA surface without dissociating from it. Thus, with increasing force, the protein can spend more time with DNA in a single sliding event, which typically slows down the target search process of proteins, explaining the lower efficiency in the non-specific search dynamics of proteins in the low-force regime.

**FIGURE 6:**
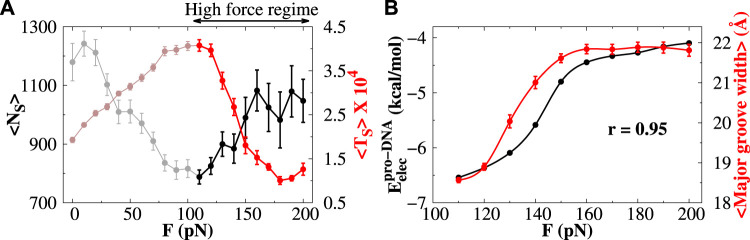
Protein diffusion under high stretching force. **(A)** Variations in the average number of sliding events ( $<N_{S}>$ ; black line) and the average time duration ( $<T_{S}>$ ; red line) of a sliding event are plotted as a function of stretching force. Only the regime under high stretching force is highlighted. **(B)** Correlations between the protein–DNA electrostatic energy and the average major groove width with the stretching force. The correlation coefficient between them is represented by *r*. The error bar for each symbol is defined as the standard error.

In contrast to the low-force regime, the average number and duration of sliding events increase and decrease, respectively, in the high-force regime (highlighted portion in Figure 6A). Unlike the low-force regime, where the electrostatic potential energy surface for triplets regulates the sliding of protein, the mechanism for protein sliding in the high-force regime is not dependent on such potential. We find that the EP energy along the DNA contour for doublets and triplets is both invariant in the high-force regime (see Supplementary Figure S10). Rather we observe that with increasing force *F*, the protein–DNA electrostatic energy $\left(E_{\mathrm{elec}}^{\mathrm{pro-DNA}}\right)$ decreases (Figure 6B, varying from more negative to less negative values). This is because with a larger applied force, the separation distance between phosphate beads increases, thus effectively weakening the protein–DNA electrostatic energy. The $E_{\mathrm{elec}}^{\mathrm{pro-DNA}}$ trend agrees well with the change in the major groove widths and shows a very strong correlation ( ∼95% , Figure 6B) with the electrostatic energy between protein and DNA (see also Supplementary Figure S11 for all force regimes). Previously, we found that such a correlation strongly influences the search dynamics of proteins while interacting non-specifically with DNA (Bhattacherjee and Levy, 2014a). Since the $E_{\mathrm{elec}}^{\mathrm{pro-DNA}}$ decreases with high force *F*, the protein cannot slide for a longer period of time, and it can either hop on DNA or can diffuse three-dimensionally in the solution. Such a combination of 1D and 3D search modes is beneficial to the fast recognition of the target DNA sites. It should be noted here that, in the high-force regime, the number of sliding events is increasing with the increase in force (highlighted portion in Figure 6A), whereas Figure 4C shows that the fraction of sliding events reduces with the increase in force. To clearly understand this behavior, we note that the fraction of sliding events and number of such events are two independent parameters and are not linked with the total number of protein diffusion events. The fraction of protein sliding is estimated by analyzing all the snapshots of the protein diffusing on DNA in a simulation trajectory. The number of snapshots in which the protein is found to perform sliding motion (see Supplementary Text for definition) is normalized with the total number of snapshots to measure the fraction of sliding. On the other hand, a sliding event is defined as the continuous snapshots of sliding motion of the protein without performing hopping or 3D diffusion dynamics. The number of snapshots in such an event is a reflection of the time duration of the event. Clearly, then for a given fraction of sliding events, if the duration of sliding events reduces, the number of sliding events rises representing short sliding events interspersed by hopping/3D diffusion dynamics. Alternatively, fewer sliding events with longer duration of each event represent smooth sliding motion of the protein along DNA.

## 4 Conclusion

While the recognition of DNA base sequences by proteins on unstretched DNA, in transcription and translation contexts, has been extensively studied in past decades, much remains to unveil about the role of extension of DNA in the nucleobase recognition by proteins. Several experimental and theoretical studies have shown that dsDNA exhibits a remarkable mechanical transition when it is stretched by an external force. When canonical B-form DNA is stretched mechanically at forces ranging 60–70 pN, its length gets elongated up to 70% to its initial contour length, and a structural transition from B-DNA into an overstretched S-DNA conformation was observed. During the transition, the existence of an overstretched Σ-DNA conformation is identified recently using a laser-tweezers force-spectroscopy experiment, where the degree of extension in Σ-form DNA is observed to be 50% extended compared to that in B-DNA. Such a Σ-DNA structure is also found in the presence of recombinase proteins. This Σ-form DNA may be considered a special case of stretched S-DNA, where the bases in Σ-DNA appear in a specific arrangement. Σ-DNA consists of stacked triplets of nucleobases, and these triplets are separated by a large rise gap. The appearance of these base triplets in stretched nucleic acids might have a biological role in enhancing the recognition of base sequences, as speculated and hypothesized recently (Bosaeus et al., 2017). To understand the connection between triplet formation and base recognition by proteins, we investigate the diffusion of a protein molecule on stretched DNA. We choose a DNA length of 100 bp, which is smaller than the DNA persistence length. For DNA longer than its persistence length, the flexibility of DNA and the partition of base-pairs are expected to be more prominent, although subjected to a detailed study. Here, we use the latest 3SPN.2C coarse-grained model of DNA to characterize the DNA dynamics, where the DNA is subjected to a constant stretching force applied to both of its 3′ ends. It is important to mention here that the 3SPN.2C DNA model has not been applied previously in the context of force-induced DNA stretching. Recently, the 3SPN.2C DNA model has been applied for estimating a small-magnitude force-dependent free energy profile of nucleosome unwrapping in which a constant force was applied to each end of DNA and nucleosomal DNA was pulled from both ends until the nucleosomal DNA unwraps from the histone surface (Lequieu et al., 2016). However, the applicability of the 3SPN.2C model is not restricted to the canonical B-DNA conformation. In a recent study, we used the same DNA model to investigate the diffusion of a protein molecule on supercoiled DNA structures with different degrees of supercoiling (Mondal et al., 2022b), suggesting the generality of the DNA model. Nevertheless, we validated the suitability of the DNA model for studying force-induced extension both qualitatively and quantitatively against the available experimental observations. The constant forces are varied between 0 and 200 pN. We find that when the dsDNA is stretched under constant force, the force–strain curve exhibits a structural transition from B-DNA to S-DNA conformation *via* an intermediate Σ-DNA state. The force–strain profile is reminiscent to the previously reported experimental observations. Our coarse-grained simulation results quantitatively describe the DNA helical parameters such as twist and rise in overstretched DNA conformations, which are consistent with the previous all-atom simulation results. Moreover, the DNA base-stacking angles in 50% stretched Σ-DNA conformation match quantitatively with the values obtained from the crystal structure of the RecA–dsDNA complex. Depending on the type of base-stacking and base-pairing interactions, different local conformations are observed which are in good agreement with the local conformations obtained from atomistic simulations. Furthermore, the resulting stretched DNA conformations show the clustering of stacked nucleobases such as doublets, triplets, and quadruplets, among which the triplet structures are found to be the most stable structure among doublets and quadruplets. This result is in qualitative agreement with the experimental measurements by Bosaeus et al. (2017). When the protein diffuses one-dimensionally along DNA, it favors the formation of base triplets in all force regimes compared to its unbound form. The relative population of base triplets reduces the ruggedness of the protein–DNA binding energy surface and results in the faster diffusion of proteins. Furthermore, we also underscore the mechanistic details of the diffusion dynamics of proteins in both low- and high-force regimes. In the low- and high-force regimes, we observed completely opposite behavior in the average number of sliding events and their time durations. In the low-force regime, the number of sliding events shows a decreasing pattern with force *F*. Analyzing the electrostatic potential in the triplet containing DNA structures, we find that the base-pair triplet reduces the ruggedness of the protein–DNA electrostatic potential surface on which the protein can smoothly perform sliding dynamics and spend higher time during sliding, which usually retards the target search efficiency. In contrast, the number of sliding events exhibits an increasing trend in the high-force regime, the rationale of which arises from the increased protein–DNA electrostatic energy due to the increased separation distance between phosphate groups, which helps the protein not to slide too long on DNA, and a right blend of the combination of 1D and 3D search modes accelerates the diffusing protein for the faster recognition of DNA bases on stretched DNA. Our study provides the plausible rationale behind the advantage of the formation of DNA base triplets that can be observed under *in vitro* conditions, the knowledge of which would be beneficial to understanding crucial DNA metabolic processes. Nevertheless, suitable experimental methods are warranted to validate our results and establish a more direct relationship between DNA base-pair partitions and flow of genetic information.

## Data Availability

The raw data supporting the conclusion of this article will be made available by the authors, without undue reservation.

## References

[B1] Alberts B. Johnson A. Lewis J. Raff M. Roberts K. Walter P. (2008). Molecular biology of the cell. New York: Garland Science.

[B2] Allemand J. F. Bensimon D. Croquette V. (2003). Stretching DNA and RNA to probe their interactions with proteins. Curr. Opin. Struct. Biol. 13, 266–274. 10.1016/s0959-440x(03)00067-8 12831877

[B3] Bertucat G. Lavery R. Prévost C. (1998). A model for parallel triple helix formation by RecA: Single-single association with a homologous duplex via the minor groove. J. Biomol. Struct. Dyn. 16, 535–546. 10.1080/07391102.1998.10508268 10052612

[B4] Bhattacherjee A. Krepel D. Levy Y. (2016). Coarse-grained models for studying protein diffusion along DNA. WIREs. Comput. Mol. Sci. 6, 515–531. 10.1002/wcms.1262

[B5] Bhattacherjee A. Levy Y. (2014a). Search by proteins for their DNA target site: 1. The effect of DNA conformation on protein sliding. Nucleic Acids Res. 42, 12404–12414. 10.1093/nar/gku932 25324308 PMC4227778

[B6] Bhattacherjee A. Levy Y. (2014b). Search by proteins for their DNA target site: 2. The effect of DNA conformation on the dynamics of multidomain proteins. Nucleic Acids Res. 42, 12415–12424. 10.1093/nar/gku933 25324311 PMC4227779

[B7] Bhattacherjee A. Wallin S. (2012). Coupled folding-binding in a hydrophobic/polar protein model: Impact of synergistic folding and disordered flanks. Biophys. J. 102, 569–578. 10.1016/j.bpj.2011.12.008 22325280 PMC3274785

[B8] Blainey P. C. Luo G. Kou S. C. Mangel W. F. Verdine G. L. Bagchi B. (2009). Nonspecifically bound proteins spin while diffusing along DNA. Nat. Struct. Mol. Biol. 16, 1224–1229. 10.1038/nsmb.1716 19898474 PMC2889498

[B9] Bosaeus N. El-Sagheer A. H. Brown T. Smith S. B. Akerman B. Bustamante C. (2012). Tension induces a base-paired overstretched DNA conformation. Proc. Natl. Acad. Sci. U. S. A. 109, 15179–15184. 10.1073/pnas.1213172109 22949705 PMC3458322

[B10] Bosaeus N. Reymer A. Beke-Somfai T. Brown T. Takahashi M. Wittung-Stafshede P. (2017). A stretched conformation of DNA with a biological role? Q. Rev. Biophys. 50, e11. 10.1017/S0033583517000099 29233223

[B11] Brandani G. B. Tan C. Takada S. (2021). The kinetic landscape of nucleosome assembly: A coarse-grained molecular dynamics study. PLoS Comput. Biol. 17, e1009253. 10.1371/journal.pcbi.1009253 34314440 PMC8345847

[B12] Chen Z. Yang H. Pavletich N. P. (2008). Mechanism of homologous recombination from the RecA-ssDNA/dsDNA structures. Nature 453, 489–484. 10.1038/nature06971 18497818

[B13] Clausen-Schaumann H. Rief M. Tolksdorf C. Gaub H. E. (2000). Mechanical stability of single DNA molecules. Biophys. J. 78, 1997–2007. 10.1016/S0006-3495(00)76747-6 10733978 PMC1300792

[B14] Clementi C. Jennings P. A. Onuchic J. N. (2000a). How native-state topology affects the folding of dihydrofolate reductase and interleukin-1beta. Proc. Natl. Acad. Sci. U. S. A. 97, 5871–5876. 10.1073/pnas.100547897 10811910 PMC18526

[B15] Clementi C. Nymeyer H. Onuchic J. N. (2000b). Topological and energetic factors: What determines the structural details of the transition state ensemble and ”en-route” intermediates for protein folding? An investigation for small globular proteins. J. Mol. Biol. 298, 937–953. 10.1006/jmbi.2000.3693 10801360

[B16] Clore G. M. (2011). Exploring translocation of proteins on DNA by NMR. J. Biomol. NMR 51, 209–219. 10.1007/s10858-011-9555-8 21847629 PMC3207612

[B17] Cluzel P. Lebrun A. Heller C. Lavery R. Viovy J. L. Chatenay D. (1996). Dna: An extensible molecule. Science 271, 792–794. 10.1126/science.271.5250.792 8628993

[B18] Dahlke K. Sing C. E. (2018). Force-extension behavior of DNA in the presence of DNA-bending nucleoid associated proteins. J. Chem. Phys. 148, 084902. 10.1063/1.5016177 29495783 PMC5825231

[B19] Danilowicz C. Limouse C. Hatch K. Conover A. Coljee V. W. Kleckner N. (2009). The structure of DNA overstretched from the 5′5′ ends differs from the structure of DNA overstretched from the 3′3′ ends. Proc. Natl. Acad. Sci. U. S. A. 106, 13196–13201. 10.1073/pnas.0904729106 19666582 PMC2717110

[B20] Dans P. D. Pérez A. Faustino I. Lavery R. Orozco M. (2012). Exploring polymorphisms in B-DNA helical conformations. Nucleic Acids Res. 40, 10668–10678. 10.1093/nar/gks884 23012264 PMC3510489

[B21] De Vlaminck I. Vidic I. van Loenhout M. T. Kanaar R. Lebbink J. H. Dekker C. (2010). Torsional regulation of hRPA-induced unwinding of double-stranded DNA. Nucleic Acids Res. 38, 4133–4142. 10.1093/nar/gkq067 20197317 PMC2896508

[B22] Dey P. Bhattacherjee A. (2019a). Disparity in anomalous diffusion of proteins searching for their target DNA sites in a crowded medium is controlled by the size, shape and mobility of macromolecular crowders. Soft Matter 15, 1960–1969. 10.1039/c8sm01933a 30539954

[B23] Dey P. Bhattacherjee A. (2019b). Mechanism of facilitated diffusion of DNA repair proteins in crowded environment: Case study with human uracil DNA glycosylase. J. Phys. Chem. B 123, 10354–10364. 10.1021/acs.jpcb.9b07342 31725289

[B24] Dey P. Bhattacherjee A. (2018). Role of macromolecular crowding on the intracellular diffusion of DNA binding proteins. Sci. Rep. 8, 844. 10.1038/s41598-017-18933-3 29339733 PMC5770392

[B25] Dey P. Bhattacherjee A. (2020). Structural basis of enhanced facilitated diffusion of DNA-binding protein in crowded cellular milieu. Biophys. J. 118, 505–517. 10.1016/j.bpj.2019.11.3388 31862109 PMC6976804

[B26] Elf J. Li G. W. Xie X. S. (2007). Probing transcription factor dynamics at the single-molecule level in a living cell. Science 316, 1191–1194. 10.1126/science.1141967 17525339 PMC2853898

[B27] Esadze A. Iwahara J. (2014). Stopped-flow fluorescence kinetic study of protein sliding and intersegment transfer in the target DNA search process. J. Mol. Biol. 426, 230–244. 10.1016/j.jmb.2013.09.019 24076422 PMC8254140

[B28] Freeman G. S. Hinckley D. M. Lequieu J. P. Whitmer J. K. de Pablo J. J. (2014a). Coarse-grained modeling of DNA curvature. J. Chem. Phys. 141, 165103. 10.1063/1.4897649 25362344

[B29] Freeman G. S. Lequieu J. P. Hinckley D. M. Whitmer J. K. de Pablo J. J. (2014b). DNA shape dominates sequence affinity in nucleosome formation. Phys. Rev. Lett. 113, 168101. 10.1103/PhysRevLett.113.168101 25361282

[B30] Garai A. Mogurampelly S. Bag S. Maiti P. K. (2017). Overstretching of B-DNA with various pulling protocols: Appearance of structural polymorphism and S-DNA. J. Chem. Phys. 147, 225102. 10.1063/1.4991862 29246060

[B31] Gemmen G. J. Millin R. Smith D. E. (2006). Tension-dependent DNA cleavage by restriction endonucleases: Two-site enzymes are ”switched off” at low force. Proc. Natl. Acad. Sci. U. S. A. 103, 11555–11560. 10.1073/pnas.0604463103 16868081 PMC1520314

[B32] Givaty O. Levy Y. (2009). Protein sliding along DNA: Dynamics and structural characterization. J. Mol. Biol. 385, 1087–1097. 10.1016/j.jmb.2008.11.016 19059266

[B33] Gore J. Bryant Z. Stone M. D. Nöllmann M. Cozzarelli N. R. Bustamante C. (2006). Mechanochemical analysis of DNA gyrase using rotor bead tracking. Nature 439, 100–104. 10.1038/nature04319 16397501 PMC1440892

[B34] Gosse C. Croquette V. (2002). Magnetic tweezers: Micromanipulation and force measurement at the molecular level. Biophys. J. 82, 3314–3329. 10.1016/S0006-3495(02)75672-5 12023254 PMC1302119

[B35] Guan L. He P. Yang F. Zhang Y. Hu Y. Ding J. (2017). Sap1 is a replication-initiation factor essential for the assembly of pre-replicative complex in the fission yeast *Schizosaccharomyces pombe* . J. Biol. Chem. 292, 6056–6075. 10.1074/jbc.M116.767806 28223353 PMC5391739

[B36] Guardiani C. Cencini M. Cecconi F. (2014). Coarse-grained modeling of protein unspecifically bound to DNA. Phys. Biol. 11, 026003. 10.1088/1478-3975/11/2/026003 24685517

[B37] Harris S. A. Sands Z. A. Laughton C. A. (2005). Molecular dynamics simulations of duplex stretching reveal the importance of entropy in determining the biomechanical properties of DNA. Biophys. J. 88, 1684–1691. 10.1529/biophysj.104.046912 15626714 PMC1305225

[B38] Hinckley D. M. Freeman G. S. Whitmer J. K. de Pablo J. J. (2013). An experimentally-informed coarse-grained 3-site-per-nucleotide model of DNA: Structure, thermodynamics, and dynamics of hybridization. J. Chem. Phys. 139, 144903. 10.1063/1.4822042 24116642 PMC3808442

[B39] Iwahara J. Clore G. M. (2006a). Detecting transient intermediates in macromolecular binding by paramagnetic NMR. Nature 440, 1227–1230. 10.1038/nature04673 16642002

[B40] Iwahara J. Clore G. M. (2006b). Direct observation of enhanced translocation of a homeodomain between DNA cognate sites by NMR exchange spectroscopy. J. Am. Chem. Soc. 128, 404–405. 10.1021/ja056786o 16402815

[B41] Kolomeisky A. B. Veksler A. (2012). How to accelerate protein search on DNA: Location and dissociation. J. Chem. Phys. 136, 125101. 10.1063/1.3697763 22462896

[B42] Kosikov K. M. Gorin A. A. Zhurkin V. B. Olson W. K. (1999). DNA stretching and compression: Large-scale simulations of double helical structures. J. Mol. Biol. 289, 1301–1326. 10.1006/jmbi.1999.2798 10373369

[B43] Lai P.-Y. Zhou Z. (2003). B- to s-form transition in double-stranded dna with basepair interactions. Phys. A Stat. Mech. its Appl. 321, 170–180. 10.1016/s0378-4371(02)01776-4

[B44] Lebrun A. Lavery R. (1996). Modelling extreme stretching of DNA. Nucleic Acids Res. 24, 2260–2267. 10.1093/nar/24.12.2260 8710494 PMC145932

[B45] Lequieu J. Córdoba A. Schwartz D. C. de Pablo J. J. (2016). Tension-dependent free energies of nucleosome unwrapping. ACS Cent. Sci. 2, 660–666. 10.1021/acscentsci.6b00201 27725965 PMC5043429

[B46] Lequieu J. Schwartz D. C. de Pablo J. J. (2017). *In silico* evidence for sequence-dependent nucleosome sliding. Proc. Natl. Acad. Sci. U. S. A. 114, E9197–E9205. 10.1073/pnas.1705685114 29078285 PMC5676884

[B47] Li L. Li C. Sarkar S. Zhang J. Witham S. Zhang Z. (2012). DelPhi: A comprehensive suite for DelPhi software and associated resources. BMC Biophys. 5, 9. 10.1186/2046-1682-5-9 22583952 PMC3463482

[B48] Lin X. Leicher R. Liu S. Zhang B. (2021). Cooperative DNA looping by PRC2 complexes. Nucleic Acids Res. 49, 6238–6248. 10.1093/nar/gkab441 34057467 PMC8216278

[B49] Liu N. Bu T. Song Y. Zhang W. Li J. Zhang W. (2010). The nature of the force-induced conformation transition of dsDNA studied by using single molecule force spectroscopy. Langmuir 26, 9491–9496. 10.1021/la100037z 20178341

[B50] Luan B. Aksimentiev A. (2008). Strain softening in stretched DNA. Phys. Rev. Lett. 101, 118101. 10.1103/PhysRevLett.101.118101 18851334 PMC2890292

[B51] Maier B. Bensimon D. Croquette V. (2000). Replication by a single DNA polymerase of a stretched single-stranded DNA. Proc. Natl. Acad. Sci. U. S. A. 97, 12002–12007. 10.1073/pnas.97.22.12002 11050232 PMC17284

[B52] Manghi M. Destainville N. Palmeri J. (2012). Mesoscopic models for DNA stretching under force: New results and comparison with experiments. Eur. Phys. J. E Soft Matter 35, 110. 10.1140/epje/i2012-12110-2 23099534

[B53] Marklund E. G. Mahmutovic A. Berg O. G. Hammar P. van der Spoel D. Fange D. (2013). Transcription-factor binding and sliding on DNA studied using micro- and macroscopic models. Proc. Natl. Acad. Sci. U. S. A. 110, 19796–19801. 10.1073/pnas.1307905110 24222688 PMC3856812

[B54] Marko J. F. (2015). Biophysics of protein-DNA interactions and chromosome organization. Phys. A 418, 126–153. 10.1016/j.physa.2014.07.045 PMC4235750 25419039

[B55] Marko J. F. Siggia E. D. (1997). Driving proteins off DNA using applied tension. Biophys. J. 73, 2173–2178. 10.1016/S0006-3495(97)78248-1 9336213 PMC1181118

[B56] Mirny L. Slutsky M. Wunderlich Z. Tafvizi A. Leith J. Kosmrlj A. (2009). How a protein searches for its site on DNA: The mechanism of facilitated diffusion. J. Phys. A Math. Theor. 42, 434013. 10.1088/1751-8113/42/43/434013

[B57] Mo Y. Vaessen B. Johnston K. Marmorstein R. (1998). Structures of SAP-1 bound to DNA targets from the E74 and c-fos promoters: Insights into DNA sequence discrimination by ets proteins. Mol. Cell. 2, 201–212. 10.1016/s1097-2765(00)80130-6 9734357

[B58] Moller J. Lequieu J. de Pablo J. J. (2019). The free energy landscape of internucleosome interactions and its relation to chromatin fiber structure. ACS Cent. Sci. 5, 341–348. 10.1021/acscentsci.8b00836 30834322 PMC6396382

[B59] Mondal A. Bhattacherjee A. (2015). Searching target sites on DNA by proteins: Role of DNA dynamics under confinement. Nucleic Acids Res. 43, 9176–9186. 10.1093/nar/gkv931 26400158 PMC4627088

[B60] Mondal A. Bhattacherjee A. (2017). Understanding the role of DNA topology in target search dynamics of proteins. J. Phys. Chem. B 121, 9372–9381. 10.1021/acs.jpcb.7b08199 28926253

[B61] Mondal A. Mishra S. K. Bhattacherjee A. (2022a). Nucleosome breathing facilitates cooperative binding of pluripotency factors Sox2 and Oct4 to DNA. Biophys. J. 10.1016/j.bpj.2022.10.039 PMC9748375 36321206

[B62] Mondal A. Sangeeta Bhattacherjee A. (2022b). Torsional behaviour of supercoiled DNA regulates recognition of architectural protein Fis on minicircle DNA. Nucleic Acids Res. 50, 6671–6686. 10.1093/nar/gkac522

[B63] Nagae F. Brandani G. B. Takada S. Terakawa T. (2021). The lane-switch mechanism for nucleosome repositioning by DNA translocase. Nucleic Acids Res. 49, 9066–9076. 10.1093/nar/gkab664 34365508 PMC8450081

[B64] Nicklas R. B. (1988). The forces that move chromosomes in mitosis. Annu. Rev. Biophys. Biophys. Chem. 17, 431–449. 10.1146/annurev.bb.17.060188.002243 3293594

[B65] Niina T. Brandani G. B. Tan C. Takada S. (2017). Sequence-dependent nucleosome sliding in rotation-coupled and uncoupled modes revealed by molecular simulations. PLoS Comput. Biol. 13, e1005880. 10.1371/journal.pcbi.1005880 29194442 PMC5728581

[B66] Okazaki K. Koga N. Takada S. Onuchic J. N. Wolynes P. G. (2006). Multiple-basin energy landscapes for large-amplitude conformational motions of proteins: Structure-based molecular dynamics simulations. Proc. Natl. Acad. Sci. U. S. A. 103, 11844–11849. 10.1073/pnas.0604375103 16877541 PMC1567665

[B67] Paik D. H. Perkins T. T. (2011). Overstretching DNA at 65 pN does not require peeling from free ends or nicks. J. Am. Chem. Soc. 133, 3219–3221. 10.1021/ja108952v 21207940

[B68] Putzel G. G. Tagliazucchi M. Szleifer I. (2014). Nonmonotonic diffusion of particles among larger attractive crowding spheres. Phys. Rev. Lett. 113, 138302. 10.1103/PhysRevLett.113.138302 25302920 PMC4670031

[B69] Rief M. Clausen-Schaumann H. Gaub H. E. (1999). Sequence-dependent mechanics of single DNA molecules. Nat. Struct. Biol. 6, 346–349. 10.1038/7582 10201403

[B70] Smith S. B. Cui Y. Bustamante C. (1996). Overstretching B-DNA: The elastic response of individual double-stranded and single-stranded DNA molecules. Science 271, 795–799. 10.1126/science.271.5250.795 8628994

[B71] Taghavi A. van der Schoot P. Berryman J. T. (2017). DNA partitions into triplets under tension in the presence of organic cations, with sequence evolutionary age predicting the stability of the triplet phase - corrigendum. Q. Rev. Biophys. 50, e1. 10.1017/S0033583517000142 29233227

[B72] Tan C. Takada S. (2020). Nucleosome allostery in pioneer transcription factor binding. Proc. Natl. Acad. Sci. U. S. A. 117, 20586–20596. 10.1073/pnas.2005500117 32778600 PMC7456188

[B73] Veksler A. Kolomeisky A. B. (2013). Speed-selectivity paradox in the protein search for targets on DNA: Is it real or not? J. Phys. Chem. B 117, 12695–12701. 10.1021/jp311466f 23316873

[B74] von Hippel P. H. Berg O. G. (1989). Facilitated target location in biological systems. J. Biol. Chem. 264, 675–678. 10.1016/s0021-9258(19)84994-3 2642903

[B75] Wu L. Xiao B. Jia X. Zhang Y. Lü S. Chen J. (2007). Impact of carrier stiffness and microtopology on two-dimensional kinetics of P-selectin and P-selectin glycoprotein ligand-1 (PSGL-1) interactions. J. Biol. Chem. 282, 9846–9854. 10.1074/jbc.M609219200 17267403

[B76] Wuite G. J. Smith S. B. Young M. Keller D. Bustamante C. (2000). Single-molecule studies of the effect of template tension on T7 DNA polymerase activity. Nature 404, 103–106. 10.1038/35003614 10716452

[B77] Xiao B. Johnson R. C. Marko J. F. (2010). Modulation of HU-DNA interactions by salt concentration and applied force. Nucleic Acids Res. 38, 6176–6185. 10.1093/nar/gkq435 20497998 PMC2952867

[B78] Xu J. Zhao L. Xu Y. Zhao W. Sung P. Wang H. W. (2017). Cryo-EM structures of human RAD51 recombinase filaments during catalysis of DNA-strand exchange. Nat. Struct. Mol. Biol. 24, 40–46. 10.1038/nsmb.3336 27941862 PMC5471492

[B79] Yin H. Wang M. D. Svoboda K. Landick R. Block S. M. Gelles J. (1995). Transcription against an applied force. Science 270, 1653–1657. 10.1126/science.270.5242.1653 7502073

[B80] Zheng G. Lu X. J. Olson W. K. (2009). Web 3DNA–a web server for the analysis, reconstruction, and visualization of three-dimensional nucleic-acid structures. Nucleic Acids Res. 37, W240–W246. 10.1093/nar/gkp358 19474339 PMC2703980

