# Supplementary material for: Understanding protein diffusion on force-induced stretched DNA conformation
Source: Front Mol Biosci. 2022 Dec 5;9:953689. doi: 10.3389/fmolb.2022.953689 (PMC9760818; doi:10.3389/fmolb.2022.953689)
Supplement: Supplementary file 1 [file DataSheet1.pdf]

Supplementary Material

1 PROTEIN MODEL

The protein is modeled through a coarse-grained (CG) representation where each protein residue is represented by one CG particle placed at the respective C_α position Bhattacherjee et al. (2016). The protein maintains its folded structure through a native topology-based model in which a Lennard-Jones potential encourages the formation of native contacts found in the crystal structure, thus help to preserve the protein native fold during the simulations Clementi et al. (2000b). Such reduced model promise great advantages in studying folding of proteins Clementi et al. (2000a), protein-protein interactions Bhattacherjee and Wallin (2012) and multiple basin free energy landscape for large scale motion of proteins Okazaki et al. (2006). In addition, electrostatic interactions between positively charged (Arg, Lys) and negatively charged (Asp, Glu) amino acids are modeled through Debye-Hückel potential. Note that Debye-Hückel theory is inadequate to capture the effect of ion condensation and is valid only for dilute solutions. Despite several limitations, the Debye-Hückel potential has successfully been applied to understand many crucial aspects of nucleic acid biophysics Mondal and Bhattacherjee (2015); Bhattacherjee and Levy (2014a,b); Dey and Bhattacherjee (2018, 2019a,b, 2020); Mondal et al. (2022a,b).

The potential energy function for protein is designated as:

$$E^{protein}_{pot} = E^{protein}_{bond} + E^{protein}_{bend} + E^{protein}_{torsion} + E^{protein}_{Native-Contacts} + E^{protein}_{ev} + E^{protein}_{elec} \quad \text{(S1)}$$

The bond energy, $E^{protein}_{bond}$ between two successive C_α beads is given by,

$$E^{protein}_{bond} = \sum_i K_b(r_i - r_i^0)^2 \quad \text{(S2)}$$

where $K_b = 100$ kJ/mol/Å^2, r_i and r_i^0 are the distances between i^{th} and $(i+1)^{th}$ C_α beads in intermediate and folded structures of the protein respectively.

The bend energy, $E^{protein}_{bend}$ for any variation in angle is given by,

$$E^{protein}_{bend} = \sum_i K_\theta(\theta_i - \theta_i^0)^2 \quad \text{(S3)}$$

where $K_\theta = 20$ kJ/mol/rad^2, θ_i and θ_i^0 are the angles between a vector connecting i^{th} and $(i+1)^{th}$ C_α atoms and a vector connecting $(i+1)^{th}$ and $(i+2)^{th}$ C_α atoms in the intermediate and folded structures of the protein respectively.

The potential energy function for torsional angle, $E^{protein}_{torsion}$ due to rotation in the dihedral angles between four consecutive C_α atoms connected by bonds is given as,

$$E^{protein}_{torsion} = \sum_i K_{\phi_1}[1 - cos3(\phi_i - \phi_i^0)] + K_{\phi_2}[1 - cos(\phi_i - \phi_i^0)] \quad \text{(S4)}$$

where $K_{\phi_1} = 0.5$ kJ/mol, $K_{\phi_2} = 1.0$ kJ/mol, ϕ_i and ϕ_i^0 are the torsional angles between i^{th}, $(i+1)^{th}$, $(i+2)^{th}$ and $(i+3)^{th}$ C_α beads in the intermediate and folded structures of protein respectively.

$E^{protein}_{Native-Contacts}$ is the conformational energy that favours the formation of the native contacts found in the folded structure and such structure-based potential (as originally proposed by Clementi *et al.* Clementi et al. (2000b)) is modeled by a Lennard-Jones potential given by,

$$E^{protein}_{Native-Contacts} = \sum_{|i-j|>3}^{native-contacts} \epsilon_{ij} \left[5\left(\frac{\sigma_{ij}}{r_{ij}}\right)^{12} - 6\left(\frac{\sigma_{ij}}{r_{ij}}\right)^{10} \right] \tag{S5}$$

where $\epsilon_{ij} = 4.18$ kJ/mol. σ_{ij} is the $C_\alpha - C_\alpha$ distance between $i - j$ pairs that are in contact with each other in the crystal structure and r_{ij} is the same distance in the intermediate structures generated during the simulations.

All the non-bonded and non-native C_α pairs are allowed to interact through short-range repulsive excluded volume interactions, $E^{protein}_{ev}$ given by,

$$E^{protein}_{ev} = \sum_{|i-j|>3}^{\substack{non-native\\contacts}} \epsilon_{ij} \left(\frac{\sigma_{ij}}{r_{ij}}\right)^{12} \tag{S6}$$

where $\epsilon_{ij} = 1.0$ kJ/mol, r_{ij} denotes the distance between i^{th} and j^{th} C_α beads and σ_{ij} is the sum of the radii of the interacting particles. The repulsion radius of the C_α bead is set to 2.0 Å.

The Debye-Hückel screened electrostatic potential energy function is given by,

$$E^{protein}_{elec} = \sum_{i<j}^{n_{elec}} \frac{q_i q_j e^{-r_{ij}/\lambda_D}}{4\pi\epsilon_0 \epsilon(T,C) r_{ij}} \tag{S7}$$

where q_i and q_j are the charges on i^{th} and j^{th} beads, r_{ij} denotes the separation between them, λ_D is the Debye screening length, ϵ_0 is the dielectric permittivity of the vacuum and $\epsilon(T,C)$ is the dielectric permittivity of the solution.

The dielectric permittivity $\epsilon(T,C)$ is a function of salt molarity C and temperature T and can be expressed as the product of their individual contributions,

$$\epsilon(T,C) = \epsilon(T)a(C) \tag{S8}$$

where

$$\epsilon(T) = 249.4 - 0.788T/K + 7.20 \times 10^{-4}(T/K)^2 \tag{S9}$$

$$a(C) = 1.00 - 2.551C/M + 5.151 \times 10^{-2}(C/M)^2 - 6.889 \times 10^{-3}(C/M)^3 \tag{S10}$$

The Debye screening length can be written as

$$\lambda_D = \sqrt{\frac{\epsilon_0 \epsilon(T,C)}{2\beta N_A e_c^2 I}} \tag{S11}$$

where $\beta = \frac{1}{k_B T}$ is the inverse thermal energy, N_A is the Avogadro's number, e_c is the elementary charge and I is the ionic strength of the solution.

2 DNA MODEL

In this study, we adopted 3SPN.2C coarse-grained model of DNA developed in de Pablo's group, where each nucleotide is described as three beads: one for the phosphate, one for the sugar and one for the base Hinckley et al. (2013); Freeman et al. (2014). The model accurately estimates the structural properties such as, major and minor groove widths which are in good aggrement with experimental results. The model successfully reproduces DNA persistence length and predicts DNA melting temperatures which are consistent with experimental results. Most importantly, the model has been successful to incorporate sequence-dependent curvature and sequence-dependent flexibility of DNA Freeman et al. (2014). These features make it a suitable candidate to study the DNA dynamics at the molecular level.

The potential energy function used to model the DNA is given by

$$E_{pot}^{DNA} = E_{bonded}^{DNA} + E_{non-bonded}^{DNA} \tag{S12}$$

E_{bonded}^{DNA} is a combination of bond, angle and torsional potentials to preserve the DNA initial structure, given by

$$\begin{aligned} E_{bonded}^{DNA} &= E_{bond}^{DNA} + E_{bend}^{DNA} + E_{torsion}^{DNA} \\ &= \sum_i K_b(r_i - r_i^0)^2 + 100K_b(r_i - r_i^0)^4 \\ &+ \sum_i K_\theta(\theta_i - \theta_i^0)^2 \\ &+ \sum_i -K_\phi exp\left(\frac{-(\phi_i - \phi_i^0)^2}{2\sigma_{\phi,i}^2}\right) \end{aligned} \tag{S13}$$

where $K_b = 0.6$ kJ/mol/Å^2 and r_i, r_i^0 are the instantaneous and equilibrium bond lengths for the ith DNA bond respectively; K_θ represent the force constant for bending which is dependent on the sequence of DNA. The sequence dependent force constant for bending energy at each base-step is shown in supplementary Table S1 that introduce the sequence-dependent flexibility of DNA. θ_i^0 and θ_i are the equilibrium and instantaneous bend angles for bend i. $K_\phi = 7.0$ kJ/mol/rad^2 and $\sigma_{\phi,i}$, ϕ_i^0 are the Gaussian well depth and equilibrium angle for the ith dihedral respectively. In this model, the dihedral forces only act on the backbone of the system, i.e., the dihedrals are formed by phosphate and sugar sites (S−P−S−P and P−S−P−S).

Additionally, a weak dihedral potential is applied to the DNA backbone which is of the form

$$E_{\phi,periodic} = K_{\phi,periodic}\left[1 + cos\left(\phi_i - \phi_i^0\right)\right] \tag{S14}$$

where $K_{\phi,periodic} = 2.0$ kJ/mol/rad^2. This energy function will provide extra stability to the helix and prevent severe deviations from the equilibrium structures.

For non-bonded potential, we use excluded volume interactions, electrostatic interactions, and base-pairing interactions as given by,

$$E_{non-bonded}^{DNA} = E_{ev}^{DNA} + E_{elec}^{DNA} + E_{bstk}^{DNA} + E_{bp}^{DNA} + E_{cstk}^{DNA} \tag{S15}$$

The excluded volume interactions, E_{ev}^{DNA} between sites i and j are modeled through a purely repulsive Lennard-Jones potential,

$$E_{ev}^{DNA} = \sum_{i<j} \begin{cases} \epsilon_r \left[\left(\frac{\sigma_{ij}}{r_{ij}} \right)^{12} - 2 \left(\frac{\sigma_{ij}}{r_{ij}} \right)^{6} \right] + \epsilon_r & r_{ij} < r_c \\ 0 & r_{ij} \geq r_c \end{cases} \tag{S16}$$

where $\epsilon_r = 1.0$ kJ/mol and $r_{ij} = \sigma_{ij}$ is the average diameter of the interacting particles. The potential only acts between those sites that are not involved in any bonded interactions or base pair non-bonded interactions.

The electrostatic interactions E_{elec}^{DNA} between all charged phosphate atoms, which are not from neighbouring nucleotides, are modeled using Debye-Hückel potential energy function given in equation S7. The phosphates are assigned a negative charge of 0.6 instead of 1.0 in order to consider the counter-ion condensation.

The base-pairing interactions can be divided into three parts: base-stacking (E_{bstk}^{DNA}), base-pair (E_{bp}^{DNA}) and cross-stacking (E_{cstk}^{DNA}) interactions. These three interactions rely on a Morse potential of the form

$$U_{Morse}(\epsilon_{ij}, \alpha_{ij}, r_{ij}) = \epsilon_{ij}(1 - e^{(-\alpha_{ij}(r_{ij} - r_{ij}^0))})^2 - \epsilon_{ij} \tag{S17}$$

which can be decomposed into a repulsive component

$$U_{Morse}^{rep}(\epsilon_{ij}, \alpha_{ij}, r_{ij}) = \begin{cases} \epsilon_{ij}(1 - e^{(-\alpha_{ij}(r_{ij} - r_{ij}^0))})^2 & r_{ij} < r_{ij}^0 \\ 0 & r_{ij} \geq r_{ij}^0 \end{cases} \tag{S18}$$

and an attractive component

$$U_{Morse}^{attr}(\epsilon_{ij}, \alpha_{ij}, r_{ij}) = \begin{cases} -\epsilon_{ij} & r_{ij} < r_{ij}^0 \\ \epsilon_{ij}(1 - e^{(-\alpha_{ij}(r_{ij} - r_{ij}^0))})^2 - \epsilon_{ij} & r_{ij} \geq r_{ij}^0 \end{cases} \tag{S19}$$

Here ϵ_{ij} denotes the well depth of attraction between sites i and j, α_{ij} is used to control the range of attraction and r_{ij}^0 is the equilibrium distance between interacting sites.

A modulating function f is also incorporated to the angle of interaction in the base-stacking, base-pairing and cross-stacking interactions, which is of the form

$$f(K, \Delta\theta) = \begin{cases} 1 & -\frac{\pi}{2K} < \Delta\theta < \frac{\pi}{2K} \\ 1 - cos^2(K\Delta\theta) & -\frac{\pi}{K} < \Delta\theta < -\frac{\pi}{2K} \text{ or } \frac{\pi}{2K} < \Delta\theta < \frac{\pi}{K} \\ 0 & \Delta\theta < -\frac{\pi}{K} \text{ or } \Delta\theta > \frac{\pi}{K} \end{cases} \tag{S20}$$

where the modulating constant K manages the attractive cone width. With these definitions, we can fully describe the potential energy function for intra-strand base-stacking interactions as

$$E_{bstk}^{DNA} = \sum^{n_{bstk}} \begin{cases} U_{Morse}^{rep}(\epsilon_{ij}, \alpha_{BS}, r_{ij}) + f(K_{BS}, \Delta\theta_{BSij}) U_{Morse}^{attr}(\epsilon_{ij}, \alpha_{BS}, r_{ij}) & r_{ij} < r_{ij}^0 \\ f(K_{BS}, \Delta\theta_{BSij}) U_{Morse}^{attr}(\epsilon_{ij}, \alpha_{BS}, r_{ij}) & r_{ij} \geq r_{ij}^0 \end{cases} \tag{S23}$$

where $K_{BS} = 6.0$, $\alpha_{BS} = 2.0$, ϵ_{ij} gives the depth of the well of attraction between interacting sites and θ_{BS} is the angle between the vector connecting sugar and base in the $5'$ direction and the vector joining the two base atoms in the 3 $'$ direction.

The potential energy function for base-pairing interactions is given by

$$E_{bp}^{DNA} = \sum^{n_{bp}} \begin{cases} U_{Morse}^{rep}(\epsilon_{ij}, \alpha_{BP}, r_{ij}) + \frac{1}{2}(1 + cos(\Delta\phi_1))f(K_{BP}, \Delta\theta_{1ij})f(K_{BP}, \Delta\theta_{2ij})U_{Morse}^{attr}(\epsilon_{ij}, \alpha_{BP}, r_{ij}) & r_{ij} < r_{ij}^0 \\ \frac{1}{2}(1 + cos(\Delta\phi_1))f(K_{BP}, \Delta\theta_{1ij})f(K_{BP}, \Delta\theta_{2ij})U_{Morse}^{attr}(\epsilon_{ij}, \alpha_{BP}, r_{ij}) & r_{ij} \geq r_{ij}^0 \end{cases} \tag{S21}$$

where $K_{BP} = 12.0$, $\alpha_{BP} = 2.0$ and f modulates the decomposition of attractive and repulsive portions of the Morse potential. The base pairing interactions is modulated by f using θ_1 and θ_2, where θ_1 is the angle between the vector joining the sense-strand sugar, base and the vector joining sense-strand base with its complementary base, while θ_2 is the same angle but on the opposite strand of DNA. The deviations from a reference dihedral angle is penalized by $\Delta\phi_1 = \phi_1 - \phi_1^0$, where ϕ_1 is the dihedral between the sugar and base on the sense and anti-sense strands of DNA.

The potential energy function for cross stacking interactions is given by

$$E_{cstk}^{DNA} = \sum^{n_{cstk}} f(K_{BP}, \Delta\theta_{3ij})f(K_{CS}, \Delta\theta_{CSij})U_{Morse}^{attr}(\epsilon_{ij}, \alpha_{CS}, r_{ij}) \tag{S22}$$

where $K_{CS} = 8.0$, $\alpha_{CS} = 4.0$. The cross stacking interaction is modulated by θ_3 and θ_{CS}, where θ_3 is the angle between the vectors connecting the sugars to the bases in a W$-$C base pair and θ_{CS} is the vector connecting the sugar to the base, and the vector connecting the base on the anti-sense strand in the $5'$ to the base in the $3'$ direction on the sense strand.

3 CALCULATION OF RUGGEDNESS OF THE CHEMICAL POTENTIAL LANDSCAPE

In the present study, the ruggedness of the potential energy landscape is calculated directly from the simulation trajectories using the method primarily adopted by Putzel *et al.* Putzel et al. (2014)and is discussed briefly here. We partition the whole simulation box into cubic cells of dimension 50 Å^3. From our simulation trajectories, we first consider the position of the center of mass of recognition region of the diffusing protein and then calculate the probability (p_i^{cell}) of occupying i^{th} cell by the searching protein. When the diffusing protein moves from i^{th} cell to j^{th} cell, then the corresponding change in free energy is given by $-k_B T ln(p_i^{cell}/p_j^{cell})$. This also signifies the change in chemical potential between these cells. Consequently, the ruggedness of the chemical potential energy landscape can be calculated, in units of $k_B T$, with the formula given by

$$\sigma(\mu^{cell}) = \sqrt{\frac{1}{N_{cells}} \sum_i \left(\ln\left(p_i^{cell}\right) - \overline{\ln\left(p_i^{cell}\right)} \right)^2} \tag{S23}$$

where N_{cells} is the number of cubic cells. This calculation is performed at each stretching force and the result is presented in Figure 4A in the main text.

4 CALCULATION OF ELECTROSTATIC POTENTIAL ENERGY ALONG DNA CONTOUR FROM DELPHI PROGRAM

We calculate electrostatic potentials along the DNA contour using DelPhi program by solving non-linear Poisson-Boltzmann Equation Li et al. (2012). Several input parameters are required for estimating electrostatic potential in and around the DNA. For instance, partial charges and atomic radii of each coarse-grained DNA beads are chosen from de Pablo's model Hinckley et al. (2013) for the calculation. The dielectric constants for the internal surface of the solvent molecules and for the exterior aqueous solution are assigned a standard value of $\epsilon_{indi} = 2$ and $\epsilon_{exdi} = 80$ respectively. Radius of the probe molecule (prbrad) is set to 1.4 Å to define the solvent accessible surface area. The ionic strength is set to a physiological concentration of 140 mM. The scale for grid spacing is set to 2 grids/Å with Debye-Hückel boundary conditions and 90% of the lattice filled (perfil).

The electrostatic potential is calculated at a reference point i along the DNA. The reference point i is chosen to be the geometric midpoint between the phosphate atoms of nucleotide i in the $5'$-$3'$ direction and nucleotide $i-2$ in the $3'$-$5'$ direction of the DNA strand. We choose two different DNA conformations at 5×10^8 and 8×10^8 MD steps from the same simulation trajectory at any given force and calculated the electrostatic potentials along these two conformations using the above procedure. Then we identified the base pairs that are involved in forming doublets and triplets along the DNA and extracted the values of the corresponding electrostatic potential energies separately from these two conformations. Finally, we take the average contribution of these two conformations for all the base doublets and triplets along the DNA and the corresponding electrostatic potential (EP, in units of k_BT per charge) is presented in Figure 5C in the main text and in Figure S6.

5 3D DIFFUSION, SLIDING, HOPPING AND 1D DIFFUSION COEFFICIENT

We analyze all the simulation trajectories at each force to quantify the propensities of different modes of diffusion such as sliding, hopping and 3D diffusion using the method adopted in our previous work Mondal and Bhattacherjee (2015); Dey and Bhattacherjee (2018) and is described briefly here. If the distance between the centers of the recognition helix and its closest DNA base pair is more than 30 Å, then the corresponding snapshots are characterized as fully unbound state where the protein diffuses three dimensionally in the bulk (3D diffusion). A snapshot is considered to be a sliding motion if it satisfy the following three conditions simultaneously: (i) centers of the protein's recognition region and the closest DNA base pair from it should be at a distance within 15 Å, (ii) at least 70% of the recognition helix should stay inside the DNA major groove and (iii) the orientation angle of the protein relative to DNA must be less than 25°. If any of these three conditions fails to satisfy and the protein is still close enough to the DNA surface, then we consider the corresponding event as hopping mode. Therefore, while scanning the DNA non-specifically, protein can exhibit either sliding or hopping motion along the DNA. This is a 1D diffusion along the DNA contour. We calculate 1D Diffusion coefficient (D_1) from the linear behavior of the mean square displacement of the center of mass of recognition helix of the protein diffusing along the stretched DNA through sliding and hopping dynamics only.

6 PROBABILITY CALCULATION OF THE FORMATION OF BASE PAIR CLUSTERS

To calculate the probability of forming different base pair clusters (i.e, doulets, triplets, quadruplets and so on), we first evaluated the distance between consecutive DNA bases, which usually represents the DNA rise per base pair. If the two consecutive nucleobases are separated by a rise gap of length 5 Å, then it

is identified as doublets. Similarly, triplets are identified if the three consecutive bases satisfy the above condition. Likewise, the formation of quadruplet, quintuplet, sextuplet, septuplet, octuplet, nonuplet and decuplet can easily be identified. By analysing a complete simulation trajectory, the total number (N_k, $k = 2, 3, 4, \ldots, 10$) of different base pair clusters can easily be counted throughout the simulation. Finally, the probability of forming doublets (p_2), triplets (p_3), quadruplets (p_4) and so on, can be calculated as

$$p_k = \frac{N_k}{\sum_{k=2}^{10} N_k}, k = 2, 3, 4, \ldots, 10 \quad \text{(S24)}$$

REFERENCES

Bhattacherjee, A., Krepel, D., and Levy, Y. (2016). Coarse-grained models for studying protein diffusion along DNA. *Wiley Interdiscip. Rev.: Comput. Mol. Sci.* 6, 515–531

Bhattacherjee, A. and Levy, Y. (2014a). Search by proteins for their DNA target site: 1. The effect of DNA conformation on protein sliding. *Nucleic Acids Res* 42, 12404–12414

Bhattacherjee, A. and Levy, Y. (2014b). Search by proteins for their DNA target site: 2. The effect of DNA conformation on the dynamics of multidomain proteins. *Nucleic Acids Res* 42, 12415–12424

Bhattacherjee, A. and Wallin, S. (2012). Coupled folding-binding in a hydrophobic/polar protein model: impact of synergistic folding and disordered flanks. *Biophys J* 102, 569–578

Chen, Z., Yang, H., and Pavletich, N. P. (2008). Mechanism of homologous recombination from the RecA-ssDNA/dsDNA structures. *Nature* 453, 489–484

Clementi, C., Jennings, P. A., and Onuchic, J. N. (2000a). How native-state topology affects the folding of dihydrofolate reductase and interleukin-1beta. *Proc Natl Acad Sci U S A* 97, 5871–5876

Clementi, C., Nymeyer, H., and Onuchic, J. N. (2000b). Topological and energetic factors: what determines the structural details of the transition state ensemble and ”en-route” intermediates for protein folding? An investigation for small globular proteins. *J Mol Biol* 298, 937–953

Dey, P. and Bhattacherjee, A. (2018). Role of Macromolecular Crowding on the Intracellular Diffusion of DNA Binding Proteins. *Sci Rep* 8, 844

Dey, P. and Bhattacherjee, A. (2019a). Disparity in anomalous diffusion of proteins searching for their target DNA sites in a crowded medium is controlled by the size, shape and mobility of macromolecular crowders. *Soft Matter* 15, 1960–1969

Dey, P. and Bhattacherjee, A. (2019b). Mechanism of Facilitated Diffusion of DNA Repair Proteins in Crowded Environment: Case Study with Human Uracil DNA Glycosylase. *J Phys Chem B* 123, 10354–10364

Dey, P. and Bhattacherjee, A. (2020). Structural Basis of Enhanced Facilitated Diffusion of DNA-Binding Protein in Crowded Cellular Milieu. *Biophys J* 118, 505–517

Freeman, G. S., Hinckley, D. M., Lequieu, J. P., Whitmer, J. K., and de Pablo, J. J. (2014). Coarse-grained modeling of DNA curvature. *J Chem Phys* 141, 165103

Hinckley, D. M., Freeman, G. S., Whitmer, J. K., and de Pablo, J. J. (2013). An experimentally-informed coarse-grained 3-Site-Per-Nucleotide model of DNA: structure, thermodynamics, and dynamics of hybridization. *J Chem Phys* 139, 144903

Li, L., Li, C., Sarkar, S., Zhang, J., Witham, S., Zhang, Z., et al. (2012). DelPhi: a comprehensive suite for DelPhi software and associated resources. *BMC Biophys* 5, 9

Mondal, A. and Bhattacherjee, A. (2015). Searching target sites on DNA by proteins: Role of DNA dynamics under confinement. *Nucleic Acids Res* 43, 9176–9186

Mondal, A., Mishra, S. K., and Bhattacherjee, A. (2022a). Nucleosome breathing facilitates cooperative binding of pluripotency factors Sox2 and Oct4 to DNA. *Biophys J*

Mondal, A., Sangeeta, and Bhattacherjee, A. (2022b). Torsional behaviour of supercoiled DNA regulates recognition of architectural protein Fis on minicircle DNA. *Nucleic Acids Res* 50, 6671–6686

Okazaki, K., Koga, N., Takada, S., Onuchic, J. N., and Wolynes, P. G. (2006). Multiple-basin energy landscapes for large-amplitude conformational motions of proteins: Structure-based molecular dynamics simulations. *Proc Natl Acad Sci U S A* 103, 11844–11849

Putzel, G. G., Tagliazucchi, M., and Szleifer, I. (2014). Nonmonotonic diffusion of particles among larger attractive crowding spheres. *Phys Rev Lett* 113, 138302

Taghavi, A., van der Schoot, P., and Berryman, J. T. (2017). DNA partitions into triplets under tension in the presence of organic cations, with sequence evolutionary age predicting the stability of the triplet phase. *Q Rev Biophys* 50, e15

7 SUPPLEMENTARY TABLES AND FIGURES

7.1 Tables

Table S1. Force constants (k_θ) for bending angle potential in 3SPN.2C DNA model.

Angle	Base-Step	k_θ kJ/mol/rad^2	Angle	Base-Step	k_θ kJ/mol/rad^2	Angle	Base-Step	k_θ kJ/mol/rad^2
A−S−P	AA	460	C−S−P	CA	206	S−P−S	AA	355
A−S−P	AT	370	C−S−P	CT	358	S−P−S	AT	147
A−S−P	AC	442	C−S−P	CC	278	S−P−S	AC	464
A−S−P	AG	358	C−S−P	CG	278	S−P−S	AG	368
P−S−A	AA	460	P−S−C	AC	442	S−P−S	TA	230
P−S−A	TA	120	P−S−C	TC	383	S−P−S	TT	355
P−S−A	CA	206	P−S−C	CC	278	S−P−S	TC	442
P−S−A	GA	383	P−S−C	GC	336	S−P−S	TG	273
T−S−P	TA	120	G−S−P	GA	383	S−P−S	CA	273
T−S−P	TT	460	G−S−P	GT	442	S−P−S	CT	368
T−S−P	TC	383	G−S−P	GC	336	S−P−S	CC	165
T−S−P	TG	206	G−S−P	GG	278	S−P−S	CG	478
P−S−T	AT	370	P−S−G	AG	358	S−P−S	GA	442
P−S−T	TT	460	P−S−G	TG	206	S−P−S	GT	464
P−S−T	CT	358	P−S−G	CG	278	S−P−S	GC	228
P−S−T	GT	442	P−S−G	GG	278	S−P−S	GG	165
P−S−P	All	300						

7.2 Figures

10 20 30 40 50 60
5'–GGGGGGGGGG GGGGGGGGGG AAGTAGTGAC TATGGAATTA GTGGCAGTGA TCGTAGCTGC

70 80 90 100
ATCGCGTAGA GGCATTACAT GGGGGGGGGG GGGGGGGGGG – 3'

#A 17, #G 58, #C 9, #T 16

Figure S1. The sequence of the 100 base pair DNA used in our study. The sequence holds 67% GC-content and 33% AT-content.

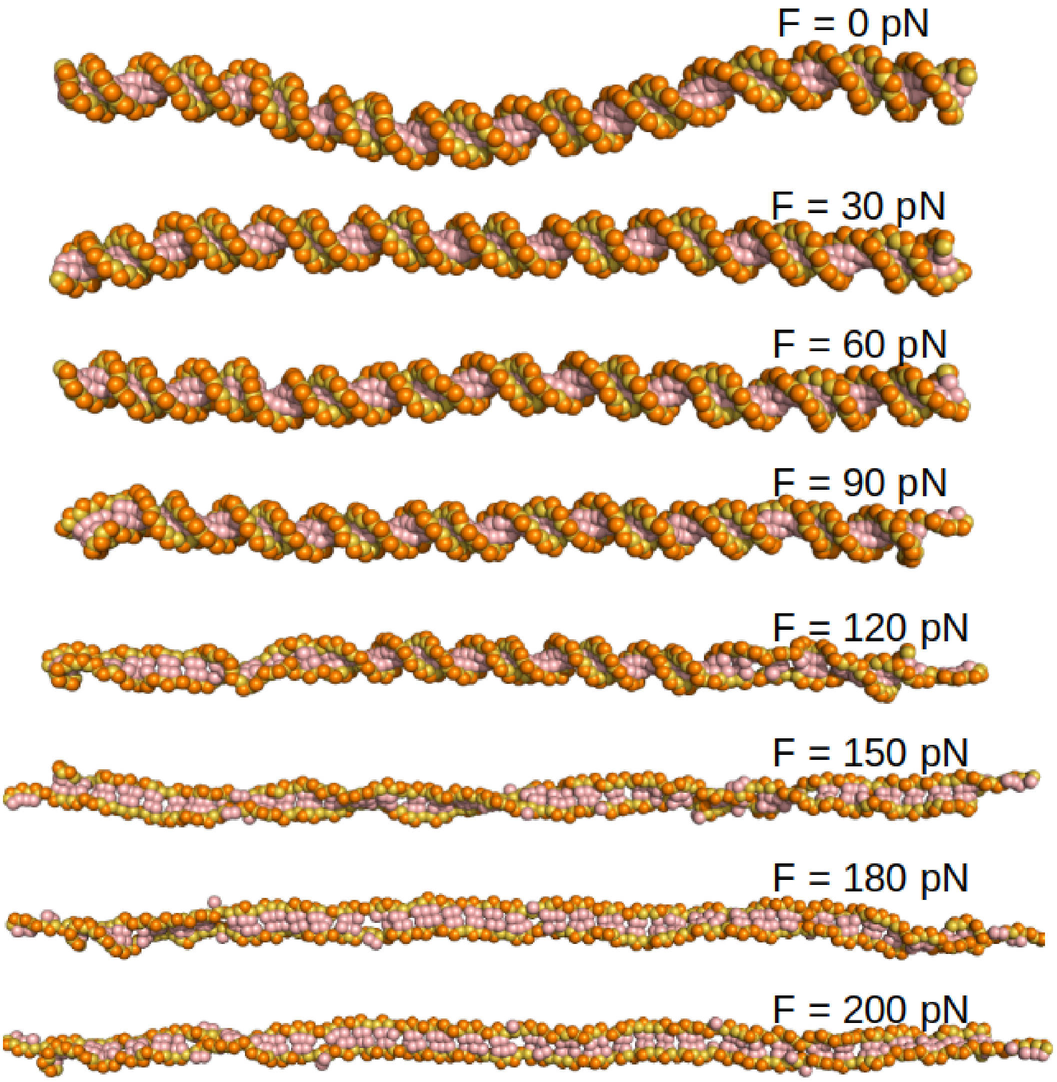

Figure S2. Snapshots of different DNA structures at various stretching force F.

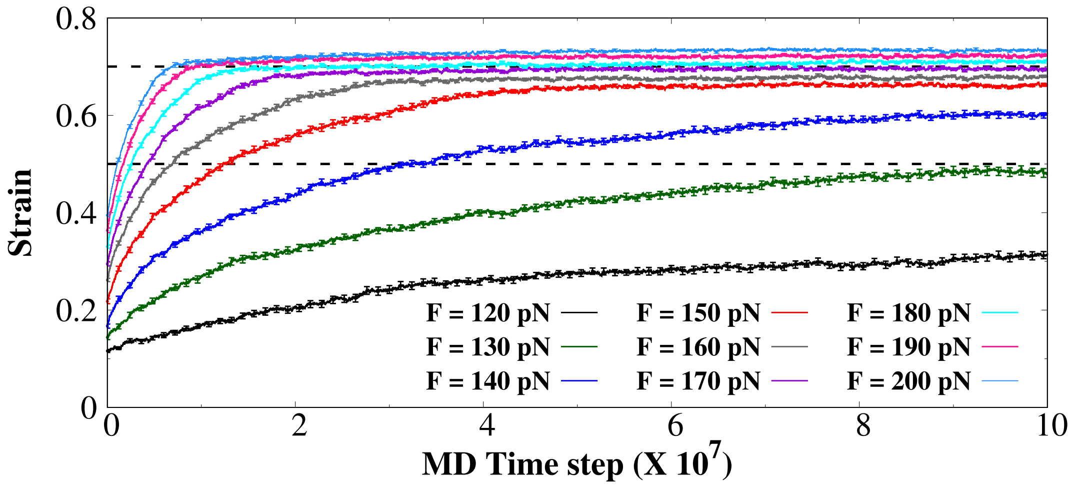

Figure S3. The kinetics of the transition between different forms of DNA at constant stretching force. The strain is presented as a function of the simulation time at forces $F = 120 - 200$ pN. The black dotted lines represent the DNA extension when it is stretched to about 50% and 70% in length compared to its initial length. The error bar is defined as the standard error.

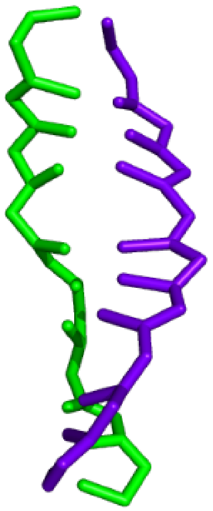

**θ_{BS} (°)**	**81.7**	**100.6**	**89.1**	**80.5**	**100.6**	**88.9**	**78.8**	**100.8**	**89.1**

Figure S4. The coarse-grained structure of the unwound stretched DNA found inside the crystal structure of the RecA-dsDNA filamentChen et al. (2008). The base stacking angles θ_{BS} between adjacent bases for the green strand are listed.

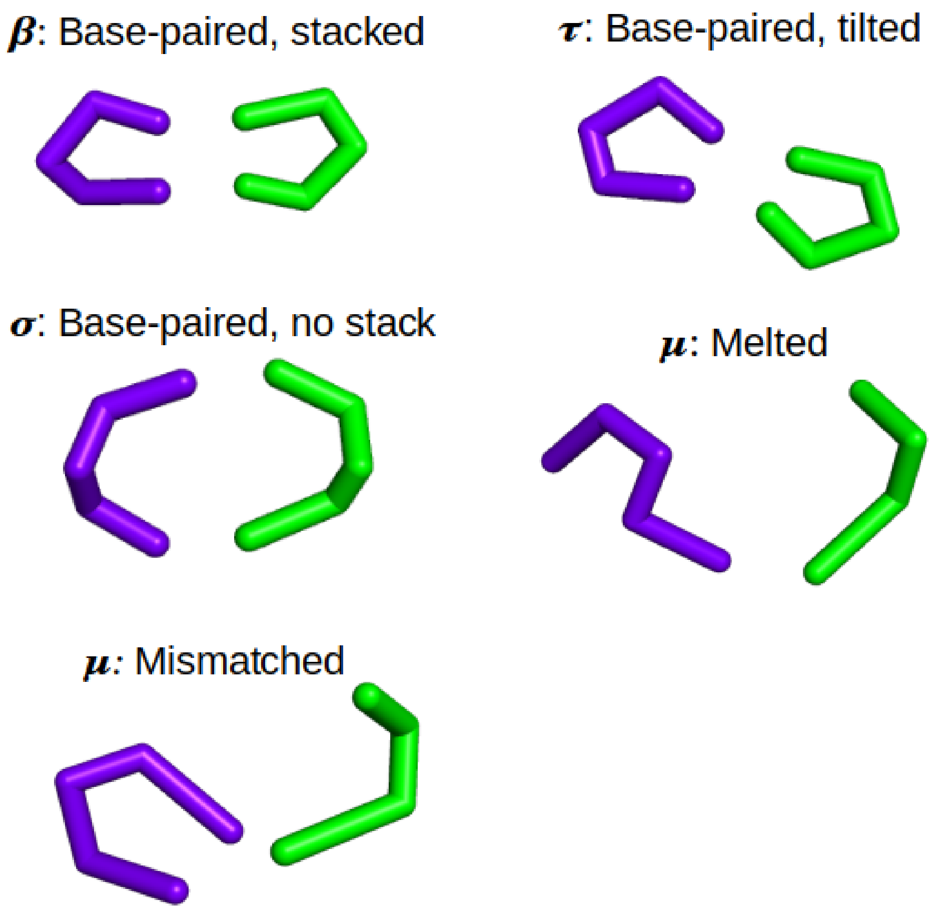

Figure S5. Different base-pair step (of sequence GG · CC) local conformations classified by the type of base-stacking and base-pairing interactions. All these local conformations are observed in the all-atom simulation of Taghavi et al. (2017) and we followed the same notation (β, τ, σ, μ) for each conformation as used in the all-atom study.

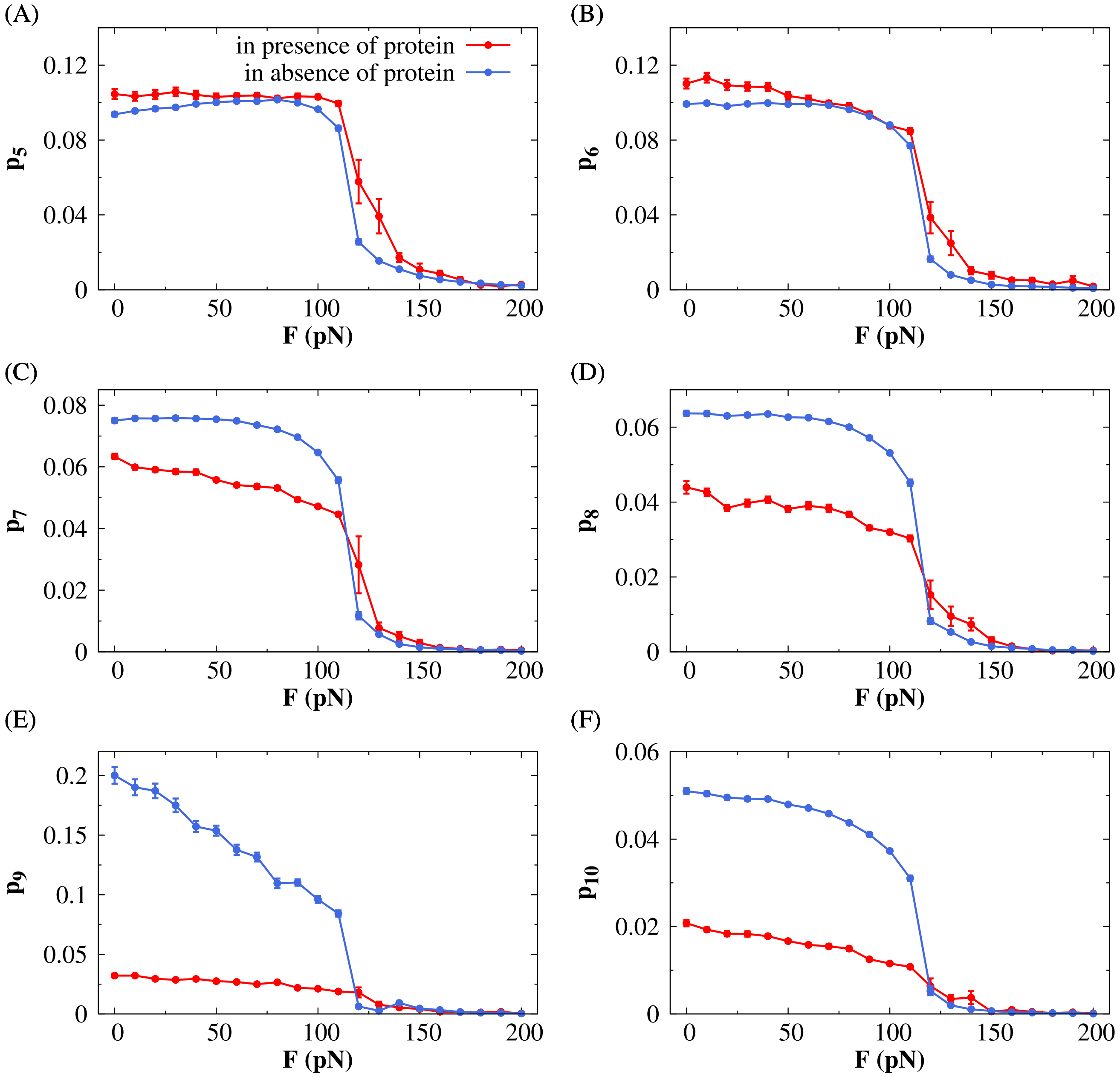

Figure S6. The probability of forming different base pair clusters as a function of stretching force in the presence (red line) and absence (blue line) of protein. Symbols p_5, p_6, p_7, p_8, p_9 and p_{10} are representing the probabilities of forming a doublet, triplet, quadruplet, quintuplet, sextuplet, septuplet, octuplet, nonuplet and decuplet respectively. The error bar for each symbol is defined as the standard error.

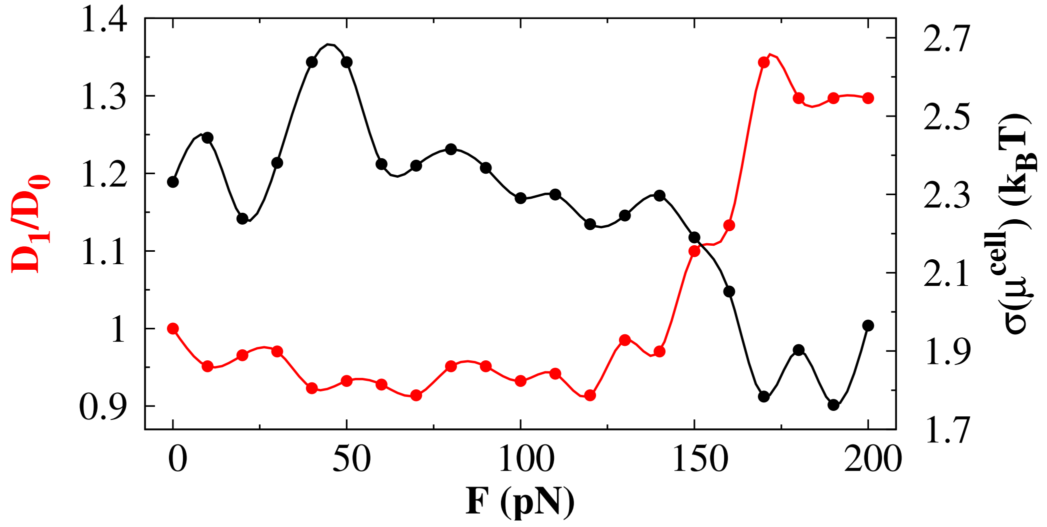

Figure S7. 1D diffusion coefficient (red line) and the ruggedness (standard deviation of μ^{cell}) of the potential energy landscape (black line) as a function of stretching force. The diffusion coefficient is divided by its value in the absence of a stretching force. The diffusion coefficient is calculated from the linear behavior of the mean square displacement of the diffusing protein through sliding and hopping dynamics. The error bar for each symbol is defined as the standard error. The associated error bars are smaller than the point size.

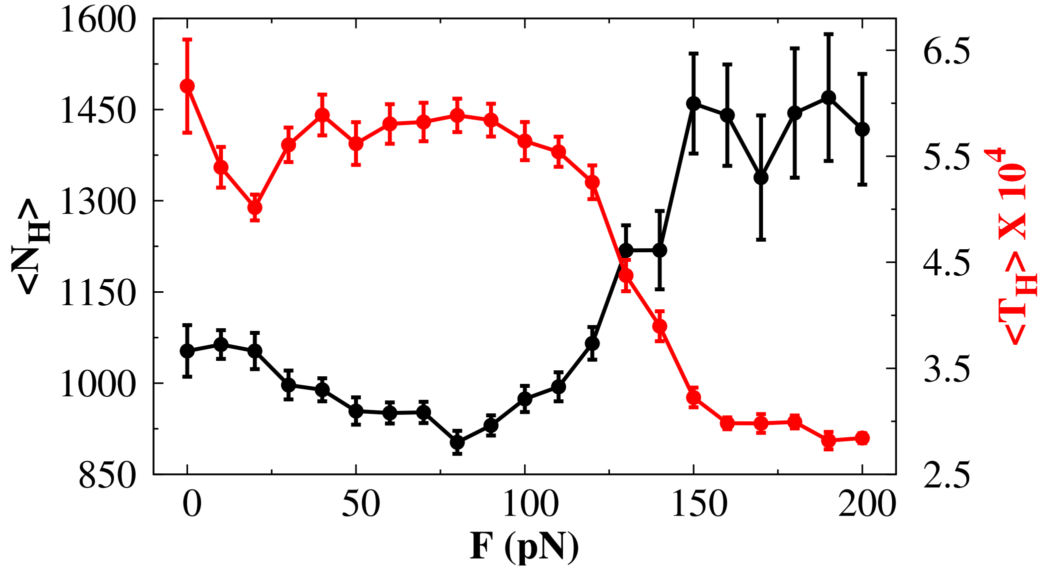

Figure S8. The variation in the average number of hopping events ($< N_H >$; black line) and the average time duration ($< T_H >$; red line) of a hopping event are plotted with respect to the constant stretching force. The error bar for each symbol is defined as the standard error.

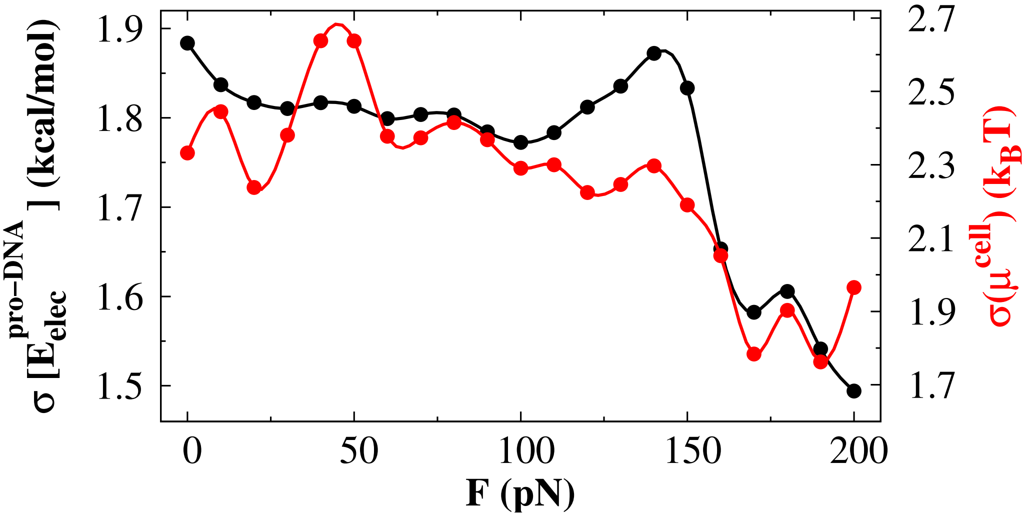

Figure S9. The fluctuation (standard deviation) in the protein−DNA electrostatic energy (black line) and the ruggedness (standard deviation of μ^{cell}) of the potential energy landscape (red line) as a function of stretching force. The error bar for each symbol is defined as the standard error. The associated error bars are smaller than the point size.

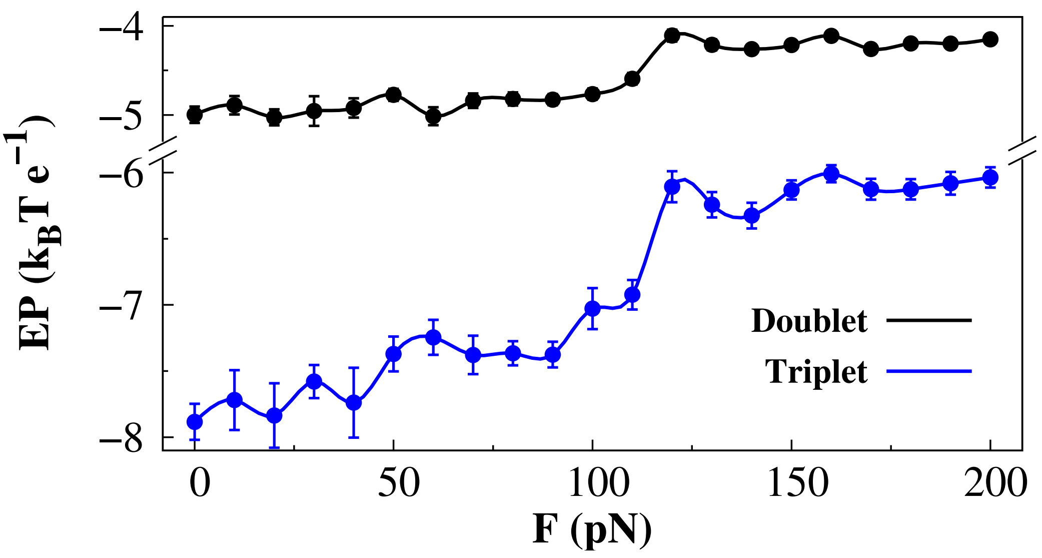

Figure S10. Electrostatic potentials along the DNA contour are calculated using DelPhi program Li et al. (2012) as a function of stretching force for doublets (black line) and triplets (blue line) along the DNA. The error bar for each symbol is defined as the standard error.

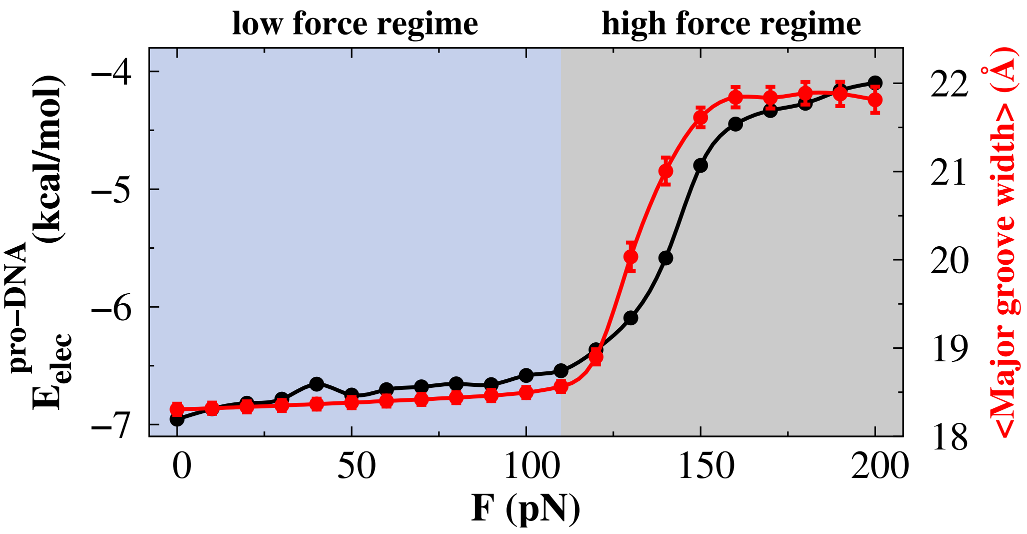

Figure S11. Correlations between the protein−DNA electrostatic energy (black line) and the average DNA major groove width (red line) under both low and high stretching force regimes. The error bar for each symbol is defined as the standard error.

8 SUPPLEMENTARY VIDEO

Supplementary Video S1: Stretching of a 100 base pair B-DNA at constant force $F = 150$ pN. The two DNA strands are shown by green and purple color.
